# Nucleolar targeting in an early-branching eukaryote suggests a general mechanism for ribosome protein sorting

**DOI:** 10.1242/jcs.259701

**Published:** 2022-10-04

**Authors:** Milad Jeilani, Karen Billington, Jack Daniel Sunter, Samuel Dean, Richard John Wheeler

**Affiliations:** ^1^ Sir William Dunn School of Pathology, University of Oxford, Oxford OX1 3RE, UK; ^2^ Department of Biological and Medical Sciences, Oxford Brookes University, Oxford OX3 0BP, UK; ^3^ Warwick Medical School, Warwick University, Warwick CV4 7AL, UK; ^4^ Peter Medawar Building for Pathogen Research, Nuffield Department of Medicine, University of Oxford, Oxford OX1 3SY, UK

**Keywords:** Nucleolus, Nucleolar targeting, Trypanosome, Mitochondrial ribosome, Liquid–liquid phase separation

## Abstract

The compartmentalised eukaryotic cell demands accurate targeting of proteins to the organelles in which they function, whether membrane-bound (like the nucleus) or non-membrane-bound (like the nucleolus). Nucleolar targeting relies on positively charged localisation signals and has received rejuvenated interest since the widespread recognition of liquid–liquid phase separation (LLPS) as a mechanism contributing to nucleolus formation. Here, we exploit a new genome-wide analysis of protein localisation in the early-branching eukaryote *Trypanosoma brucei* to analyse general nucleolar protein properties. *T. brucei* nucleolar proteins have similar properties to those in common model eukaryotes, specifically basic amino acids. Using protein truncations and addition of candidate targeting sequences to proteins, we show both homopolymer runs and distributed basic amino acids give nucleolar partition, further aided by a nuclear localisation signal (NLS). These findings are consistent with phase separation models of nucleolar formation and physical protein properties being a major contributing mechanism for eukaryotic nucleolar targeting, conserved from the last eukaryotic common ancestor. Importantly, cytoplasmic ribosome proteins, unlike mitochondrial ribosome proteins, have more basic residues – pointing to adaptation of physicochemical properties to assist segregation.

## Teaser

**Summary:** Protein targeting to the nucleolus in *Trypanosoma brucei* is mediated by positive charge, indicating a probable eukaryote-wide mechanism, and contributes to the sorting of mitochondrial from cytoplasmic ribosomes.

## INTRODUCTION

The nucleolus or nucleoli are typically the largest non-membrane bound compartments within the nucleus and are found near-universally in eukaryotes. The unicellular parasite *Trypanosoma brucei* is no exception. This early-branching eukaryote causes African trypanosomiasis (sleeping sickness) in humans and nagana in animals. The nucleolus is best known as the specialised site for ribosome biogenesis, but likely has additional functions (Dubois and Boisvert, 2016). A prerequisite for specialised function of any organelle is protein partitioning, but protein features defining targeting to the nucleolus and whether they are conserved in early-branching eukaryotes are incompletely understood.

Nucleolar targeting is of particular interest in *T. brucei* as they use RNA polymerase I (Pol I) and basal Pol I transcription factors for transcription of the major surface antigen protein-coding gene (Günzl et al., 2003; Pays et al., 1989) in addition to transcription of ribosomal RNA (rRNA) precursors. Transcription of these genes occurs in different subnuclear compartments, the expression site body (ESB) and the nucleolus, respectively (Daniels et al., 2010; Landeira and Navarro, 2007; Navarro and Gull, 2001), potentially complicating targeting of Pol I machinery. Furthermore, this changes through the life cycle, with the ESB only present in the bloodstream form parasites.

Many nuclear localisation signals (NLSs) are a short linear motif (Dingwall et al., 1982; Mattaj and Englmeier, 1998), but a similar nucleolar localisation signal (NoLS) has remained elusive. Many sequences are known to be NoLSs, typically identified through motif deletion mutants (Duan et al., 2019; Iyama et al., 2018; Musinova et al., 2011; Savada and Bonham-Smith, 2013; Schmidt-Zachmann and Nigg, 1993), and are used as the basis of predictive tools (Scott et al., 2010, 2011). Generally, NoLSs are positively charged (many basic amino acids), and it is proposed that electrostatic interactions confer nucleolar enrichment (Musinova et al., 2011, 2015; Savada and Bonham-Smith, 2013). However, high NoLS sequence diversity and distinguishing NLSs and NoLSs (both have many basic amino acids) make NoLS prediction challenging (Martin et al., 2015).

The nucleolus has liquid-like properties (Brangwynne et al., 2009, 2011; Feric et al., 2016), and recent work has shown that nucleolar proteins can undergo liquid–liquid phase separation (LLPS) *in vitro* (Boeynaems et al., 2018; Feng et al., 2019; Gomes and Shorter, 2019; Lafontaine et al., 2021; Sawyer et al., 2019b; Wang and Zhang, 2019; Woodruff et al., 2018). LLPS as a model for nucleolus formation provides a new conceptual framework for understanding nucleolar targeting (Feric et al., 2016; Lafontaine et al., 2021). In the LLPS model, key abundant components termed ‘scaffolds’ have characteristic physicochemical properties, which lead to their phase separation under cellular conditions, giving a condensate phase with an up to 100-fold higher concentration of scaffold (Li et al., 2012; Nott et al., 2015). Building on this model, the condensate is a different environment to the surrounding cytoplasm, defined by the physicochemical properties of the scaffold, and favourable interaction or solvation in the condensate allows partition of ‘client’ proteins into the condensate (Feng et al., 2019; Lafontaine et al., 2021; Martin and Holehouse, 2020).

Multivalent scaffold–scaffold interaction is important for LLPS and often arises from intrinsically disordered regions (IDRs) (Banani et al., 2017; Boeynaems et al., 2018; Duan et al., 2019; Holehouse et al., 2017; Lin et al., 2018; Stenström et al., 2020). IDRs are over-represented in membraneless organelle proteins, including nucleolar proteins, particularly those able to undergo phase separation *in vitro* (Lin et al., 2015; Martin and Mittag, 2018; Meng et al., 2015; Sawyer et al., 2019a; Stenström et al., 2020; Wright and Dyson, 2015). IDR amino acid composition tends to be better conserved than primary sequence suggesting physicochemical properties rather than simply electrostatic interactions dictate LLPS behaviour (Martin and Mittag, 2018; Weber, 2017). Growing evidence that nucleolar protein IDRs drive partition to the nucleolus phase (Duan et al., 2019; Stenström et al., 2020) might explain the lack of a specific NoLS sequence.

We suggest that study of an early-branching eukaryote like *T. brucei* will give more insight to protein partition to the nucleolus, in addition to highlighting the importance of a species-specific model. Previous analysis of NLSs in *T. brucei* have convincingly shown the canonical monopartite NLS (K-K/R-X-K/R) in model eukaryotes (Chelsky et al., 1989) is functional in *T. brucei* (Marchetti et al., 2000) and classical NLSs are strongly enriched in *T. brucei* nuclear proteins identified by mass spectrometry (Canela-Pérez et al., 2019; Goos et al., 2017). *T. brucei* and *T. cruzi* α-importin has also been shown to bind to a bipartite NLS (Afrin et al., 2020; Canela-Pérez et al., 2018, 2020). NLS conservation in such an early-branching eukaryote strongly suggests that this is the ancestral nuclear transport mechanism, and likely common across eukaryotes. We have applied similar logic to understand NoLSs.

Here, we exploit genome-wide localisation data from our *T. brucei* localisation database TrypTag (http://tryptag.org/; Dean et al., 2017), to quantify enrichment of a tagged copy of every *T. brucei* protein in the nucleus and nucleolus. Re-identification of the canonical NLS validated this approach, and it identified basic amino acids as the key protein feature associated with nucleolar partition, both in short IDRs and distributed through a protein, and importantly in an early-branching eukaryote. This shows that protein charge is the mechanism for nucleolar targeting in diverse eukaryotes, and therefore likely inherited from the last common eukaryotic ancestor, and is consistent with a LLPS model of nucleolar formation forming an environment which promotes partition of basic client proteins. Importantly, mitochondrial ribosome (mitoribosome) proteins have a distinct charge profile in comparison to cytoplasmic ribosome (cytoribosome) proteins, suggesting a contributing mechanism to their localisation.

## RESULTS

### A genome-wide map of protein partition into the nucleus and nucleolus

The TrypTag genome-wide protein localisation project (Dean et al., 2017) has generated tagged cell lines and captured high resolution microscope images of cell lines expressing endogenously tagged copies of 89% of *T. brucei* proteins (excluding variant surface glycoproteins). Protein tagging with mNeonGreen (mNG) was attempted at both the N- and C-terminus, with N- and C-terminal data available for >75% of cell lines. Each cell line was recorded through diffraction limited widefield epifluorescence images of multiple fields of view, typically four or more fields of view containing ≥250 cells to give ∼5×10^6^ cells in total.

**Fig. 1.**
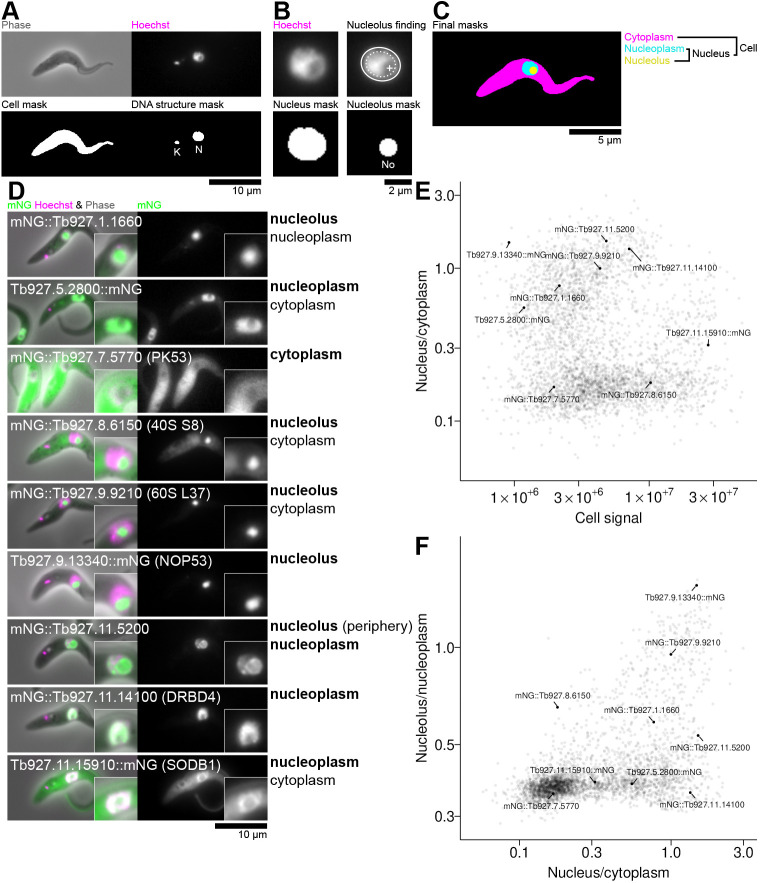
**Image analysis strategy and automated quantifcation of cytoplasm, nucleus and nucleolus signal intensity.** (A) Example input for cell, kinetoplast and nucleus identification and the resulting masks. Top, the input phase contrast and Hoechst (DNA stain) image. Bottom, the resulting cell and DNA structure masks, with the DNA-containing structures identified as the nucleus (N) and the kinetoplast (K) labelled. (B) Nucleolus identification methodology from the input nucleus Hoechst image (top left) and mask (bottom left). Mean nucleus radius is determined from the nucleus mask, here *r*=26 px. Top right, the darkest point at least *r*/8 (dotted white oval) from the edge of the nucleus (solid white oval) is taken as the nucleolus centre (plus mark). Bottom right, a circle radius *r*/4 is taken as the nucleolus. (C) The combined resulting masks. Integrated signal in the green fluorescence channels in each of these regions are used for calculation of partition. (D) mNG-tagged *T. brucei* proteins showing a range of proteins with nucleolar, nucleoplasmic and/or cytoplasmic proteins used to validate image analysis. Localisation annotations are shown next to each image, with the major (stronger signal) localisation(s) shown in bold. (E) Automated quantification of the ratio of nucleus to cytoplasm mNG fluorescence signal plotted against sum mNG fluorescence signal. The average signal and nucleus/cytoplasm ratio for all cells (typically >200 cells from one non-clonal cell line) for all cell lines in the TrypTag protein localisation database manually annotated as localising to the nucleolus, nucleoplasm and/or cytoplasm (>3000 cell lines) is plotted. The cell lines in D are labelled. (F) Automated quantification of the ratio of nucleolar to nucleoplasm mNG fluorescence signal plotted against the ratio of nucleus to cytoplasm mNG fluorescence signal, for the same cell lines as E and with the cell lines in D labelled.

We use automated high content image analysis to analyse the partition of proteins to the nucleus versus cytoplasm and partition to the nucleolus versus nucleoplasm. The nucleus was identified using signal from the DNA stain Hoechst 33342, and the nucleolus centre identified from the darkest point near the centre of the nucleus (Fig. 1A–C). Per cell, sum signal intensity from mNG fluorescence was calculated for the cytoplasm and nucleus, with nuclear signal further broken down to sum nucleoplasm or nucleolar signal intensity. Each cell was analysed individually then averaged to generate per-cell line (i.e. per N- or C-terminally tagged protein) total cell signal, nucleus/cytoplasm signal partition, and nucleolus/nucleoplasm signal partition data.

**Fig. 2.**
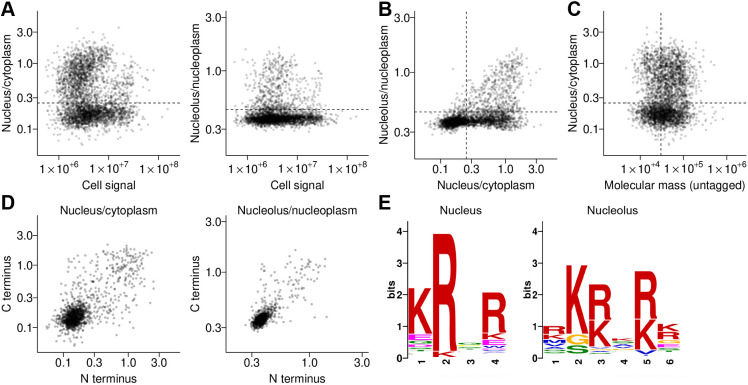
**Protein motifs in nucleus and nucleolus *T. brucei* proteins identified from automated protein localisation quantification.** (A) Thresholds for classification of proteins as nuclear or nucleolar, shown on plots of the ratio of nucleus to cytoplasm mNG fluorescence signal or the ratio of nucleolar to nucleoplasm mNG fluorescence plotted against sum mNG fluorescence signal (replotting data in Fig. 1E,F). The nucleus/cytoplasm ratio or nucleolus/nucleoplasm ratio used to classify proteins as nuclear and/or nucleolar, respectively, is shown as a horizontal dashed line in each plot. (B) Thresholds for classification of proteins as nuclear or nucleolar, shown on a plot of nucleolus/nucleoplasm ratio plotted against nuclear/cytoplasm ratio (replotting data in Fig. 1F). The vertical dashed line represents the nucleus/cytoplasm nuclear classification cutoff and the horizontal dashed line the nucleolus/nucleoplasm nucleolus classification cutoff. (C) Nucleus/cytoplasm ratio plotted against protein molecular mass, ignoring the mNG tag molecular mass. There is no clear correlation between molecular weight and nucleus/cytoplasm partition. The horizontal dashed line represents the nucleus/cytoplasm nuclear classification cutoff and the vertical dashed line an approximate cutoff (30 kDa) for protein expected to be too large to diffuse through the nuclear pore. (D) Correlation of nucleus/cytoplasm ratio and nucleolus/nucleoplasm ratio for all cell lines in the TrypTag database where the same protein tagged at either the N- or C-terminus with mNG gave signal intensity above the background intensity (>700 cell line pairs). (E) Protein motifs identified by MEME (Bailey et al., 2009) for proteins above the nucleus/cytoplasm and nucleolus/nucleoplasm cutoffs. Horizontal axes represent residue position in the motif.

To validate the quality of this analysis, we manually selected cell lines representing the diversity of cytoplasm, nucleus and nucleolus partitioning (Fig. 1D), and confirmed that the automated quantification was visually consistent with their localisation (Fig. 1E,F). Distinct populations with high nucleus/cytoplasm and high nucleolus/nucleoplasm partition were readily visible (Figs 1E,F, 2A). A weak positive correlation of nucleus/cytoplasm partition for nuclear proteins was visible – likely arising from a constant cell autofluorescence background. Note that there is a diversity in strength of partition to the nucleus and/or nucleolus, for example, from nucleus exclusion, to both nucleus and cytoplasm to nucleus only. A nuclear or nuclear localisation is not a binary classification, likely reflecting nuance in biological function. However, for ongoing analysis, we defined nucleus/cytoplasm and nucleolus/nucleoplasm partition thresholds to classify proteins as nucleolar, nucleoplasmic, nuclear (nucleolar or nucleoplasmic) or cytoplasmic (neither nucleolar nor nucleoplasmic) (Fig. 2A,B). These were selected as inclusive thresholds; for example, a tagged protein was classified as nucleolar so long as it has high nucleolar/nucleoplasm partition, but might also have easily visible nucleoplasmic and/or cytoplasmic signal.

The nuclear pore is a diffusion barrier, expected to reduce nuclear access for proteins >60 kDa. The mNG tag and linker is ∼30 kDa, therefore we might see a threshold of ∼30 kDa untagged molecular mass in nuclear/cytoplasm partition behaviour. However, we saw no clear correlation of nuclear/cytoplasm partition with molecular mass (Fig. 2C).

For many proteins, both N- and C-terminally tagged cell lines were successfully generated. We compared the nucleus/cytoplasm and nucleolus/nucleoplasm partition for N- and C-terminally tagged proteins, which showed a good positive correlation although with some outliers (Fig. 2D). These outliers might be biologically significant, for example, corresponding to the fluorescent protein sterically hindering access to a localisation sequence when on one terminus or perturbed expression level through replacement of the 3′ or 5′ UTR. However, for ongoing analysis, we took an inclusive approach treating evidence from N- or C-terminal tagging independently – essentially classifying a protein as nucleolar or nucleoplasmic if there was evidence from either terminus.

Using these nucleolar and nucleoplasmic gene lists, we searched for motifs enriched in each set using MEME (Bailey et al., 2009). This identified one statistically significant motif for each list – the canonical nuclear localisation signal (NLS) KRXR.

### KRXR is necessary and sufficient for protein targeting to the nucleus

To determine whether KRXR is necessary for protein targeting to the nucleus, we searched the genome for proteins with this motif near (contained within 15 amino acids of) either the N- or C-terminus. We selected these proteins as it is possible to remove the candidate NLS through a small open reading frame (ORF) truncation at the endogenous locus using a PCR based approach, which we validated using western blotting and sequencing of the modified loci for a subset of cell lines (Fig. S1).

**Fig. 3.**
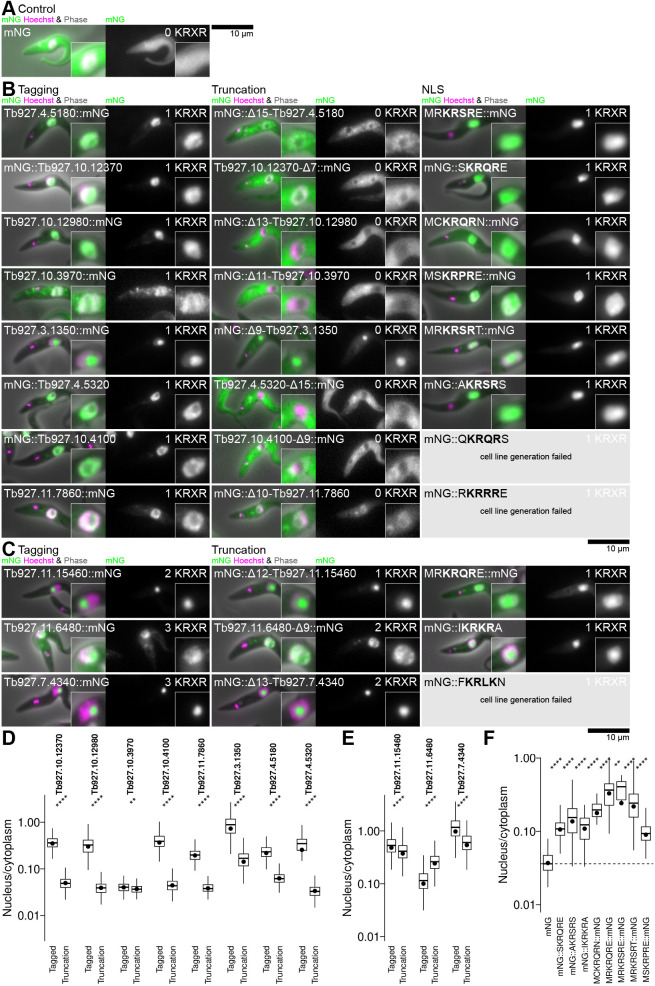
**The canonical KRXR NLS is a functional NLS that is sufficient and necessary for targeting of many proteins to the nucleus.** (A) Localisation of mNG when expressed in *T. brucei* cells. (B) Testing the candidate NLS found in eight nuclear proteins with a single candidate NLS near the N- or C-terminus. The three columns show localisation of the protein with tagging at the endogenous locus; localisation following truncation to remove the NLS and replacement with mNG; and localisation of mNG fused to the candidate NLS. For each cell line, the phase contrast, mNG fluorescence and DNA stain images are shown merged on the left and the mNG fluorescence alone on the right. The number of KRXR motifs in the fusion, accounting for truncation and NLS addition, is shown in the top right. (C) As for B, except for three nuclear proteins with multiple candidate NLSs, one of which is near the N- or C-terminus. Microscope images are from one non-clonal cell line, representative of one to four independently-generated cell lines (listed in full in Table S1). (D) Plots of automated quantification of the nucleus/cytoplasm mNG fluorescence signal partition from the cell lines in B. (E) Plots of automated quantification of the nucleus/cytoplasm mNG fluorescence signal partition from the cell lines in C. (F) Plots of automated quantification of the nucleus/cytoplasm mNG fluorescence signal partition from the cell line in A, and those successful cell lines in B and C that involve mNG fused to a candidate NLS. Quantification is from one of the independently generated cell lines, from at least 20 (on average 360) cells. The box represents the interquartile range and the median is indicated (line), whiskers represent the 5th and 95th percentile, black circles indicate the mean. ns, not significant; *P≤0.05; **P≤0.01; ***P<0.001; ****P≤0.0001 (Wilcoxon signed-rank test).

We selected eight genes with a single candidate NLS near the N- or C-terminus. Truncation of these genes to remove the NLS (and introduce an mNG tag) is a test of whether the candidate NLS is sufficient for nuclear localisation (Fig. 3A,B). All except one protein (mNG::Δ9-Tb927.3.1350) showed that the NLS was necessary for a strong nuclear localisation, although only two proteins (mNG::Δ13-Tb927.10.12980 and mNG::Δ11-Tb927.10.3970) appeared to be completely excluded from the nucleus in the absence of their NLS. We selected a further three genes with multiple candidate NLSs where only one candidate NLS is near the N- or C-terminus (Fig. 3C). We expect these N- or C-terminal NLSs not to be necessary for the nuclear localisation of the proteins, and indeed all were not necessary for a strong nuclear localisation. Overall, this is consistent with KRXR being a major nuclear targeting signal; however, alternative nuclear targeting mechanisms likely also occur (e.g. for Tb927.3.1350) and some, reduced, localisation to the nucleus can occur without the KRXR NLS.

To test whether these NLSs are sufficient to confer a nuclear localisation, we generated cell lines expressing mNG fused to the NLS from each gene, again validated using western blotting and sequencing of the modified locus for a subset of cell lines (Fig. S1). In each case, we took the NLS sequence with one flanking amino acid and fused it to the mNG at the N- or C-terminus based on where it was found in the source gene. This allows measurement of partition conferred by the targeting sequence, with presence of mNG fluorescence also confirming that the fusion is correctly folded and not degraded. With this approach, we had a low success rate at generating cell lines with an NLS fused to the mNG N-terminus. However, all successfully tested NLSs were sufficient to confer a strong nuclear localisation (Fig. 3B,C,F). Across all nuclear proteins, an arginine residue (R) was the most common residue found for the X in KRXR, most commonly flanked by arginine and glutamic acid (E) up and downstream, respectively. RKRRRE is likely an optimal NLS and we have experimentally validated RKRSRE, SKRQRE, CKRQRN, SKRPRE, RKRSRT, RKRQRE, AKRSRS and IKRKRA NLSs in *T. brucei*.

The canonical KRXR NLS is strongly enriched in nucleolar and nucleoplasmic proteins – found in 56.9% of proteins listed as nuclear by our analysis; however, presence of KRXR alone is not a good predictor of a nuclear localisation, with 55.4% of proteins with KRXR not listed as nuclear.

### A high proportion of positively charged amino acids is associated with nucleolar localisation

Motif analysis did not identify any statistically significant linear protein motifs associated with nucleolar localisation. To investigate the properties of nucleolar proteins that might confer a general mechanism for nucleolar targeting, we analysed general protein features of nucleolar proteins in comparison to nucleoplasmic proteins, nuclear (either nucleolar or nucleoplasmic) proteins and cytoplasmic proteins.

**Fig. 4.**
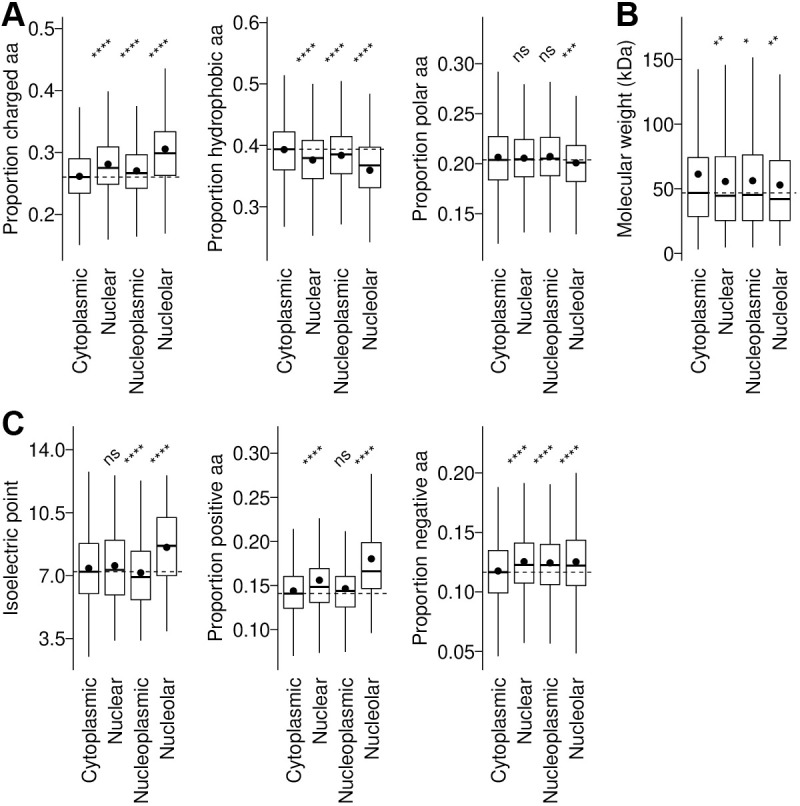
**Properties of *T. brucei* nuclear, nucleoplasmic and nucleolar proteins.** (A) Proportion of charged (RHKDE), hydrophobic (AILMFWYV) or polar (STNQ) amino acids found in cytoplasmic, nuclear, nucleoplasmic or nucleolar proteins, as classified by the cut-offs indicated in Fig. 2. (B) Molecular masses of cytoplasmic, nuclear, nucleoplasmic or nucleolar proteins. (C) Exploration of the abundance of charged amino acids in nucleolar genes shown in Fig. 2A. Isoelectric point and proportion of positively or negatively charged amino acids of cytoplasmic, nuclear, nucleoplasmic or nucleolar proteins. The box represents the interquartile range and the median is indicated (line), whiskers represent the 5th and 95th percentile, black circles indicate the mean. ns, not significant; *P≤0.05; **P≤0.01; ***P<0.001; ****P≤0.0001 (Wilcoxon signed-rank test). aa, amino acids.

Analysis of amino acid composition showed nucleolar proteins tended to have a similar proportion of polar amino acids, fewer hydrophobic amino acids and more charged amino acids in comparison to cytoplasmic proteins (Fig. 4A), whereas there was no bias in molecular mass (Fig. 4B). Further investigating the charged amino acids, nucleolar proteins were made up of a similar proportion of negatively charged (acidic) amino acids to cytoplasmic proteins but had far more positively charged (basic) amino acids, and correspondingly tended to have higher isoelectric points (Fig. 4C). Nucleolar proteins tended to have larger predicted unstructured domains, in which the basic amino acids were often found (Fig. S2).

Three other eukaryotes have comparable genome-wide protein localisation resources to *T. brucei* – the yeast species *Saccharomyces cerevisiae* and *Schizosaccharomyces pombe* (by fluorescent protein tagging) and human cell lines (by antibody). We used these to determine whether the tendency for nucleolar proteins to be highly positively charged was conserved across these species using an equivalent analysis (Fig. S3). As genome-wide quantitative analysis of nucleolar/nucleoplasm partition is not available for these species, we used the manually assigned annotation terms from each localisation project to determine whether a protein was nucleolar. This showed that, in each organism, nucleolar proteins tended to have many charged amino acids and disproportionately more positively charged amino acids, although *S. cerevisiae* and *S. pombe* also had increased numbers of negatively charged amino acids in nucleolar proteins too.

### Positively charged amino acids are sufficient for nucleolar targeting

**Fig. 5.**
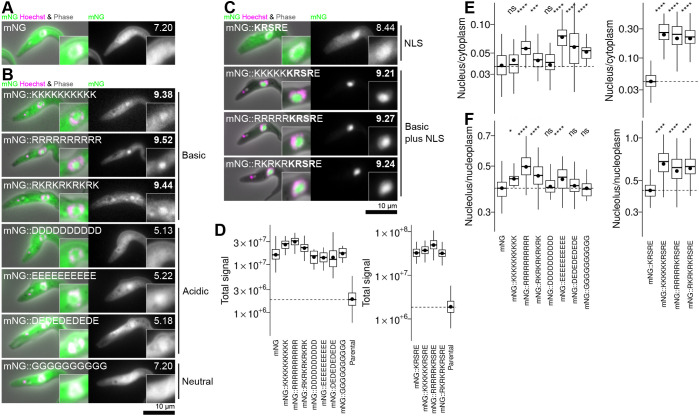
**Basic sequences are sufficient for nucleolar targeting for cytoplasmic and nuclear proteins.** (A) The localisation of the fluorescent reporter protein mNG expressed from the PFR2 locus. For each cell line, the mNG fusion protein pI is shown in the top right, as is presented in bold if >8.50. (B) The localisation of mNG with 10 amino acid runs of basic (K or R), acidic (D or E) or neutral (G) amino acids fused to the C-terminus. (C) The localisation of mNG with five basic amino acids (K, R or a mixture) and an NLS (KRSRE) fused to the C-terminus. Microscope images are from one non-clonal cell line, representative of one to three independently generated cell lines (listed in full in Table S1). (D) Plots of automated quantitation of the total mNG fluorescence signal partition from the cell lines shown in A–C, relative to the untagged parental cell line. (E) Plots of automated quantification of the nucleus/cytoplasm mNG fluorescence signal partition from the cell lines shown in A–C. (F) As for D, but plotting the nucleolus/nucleoplasm mNG fluorescence signal partition. Quantification is from one of the independently generated cell lines, from at least 20 (on average 360) cells. The box represents the interquartile range and the median is indicated (line), whiskers represent the 5th and 95th percentile, black circles indicate the mean. ns, not significant; *P≤0.05; **P≤0.01; ***P<0.001; ****P≤0.0001 (Wilcoxon signed-rank test).

mNG is a neutral (isoelectric point 7.2) globular protein with a typical proportion of negatively (25/237, 10.5%) and positively (32/237, 13.5%) charged amino acids, which localised throughout the cytoplasm and nucleus when expressed in *T. brucei* (Fig. 5A). Fusion with an NLS confers a nuclear localisation with the protein present in both the nucleoplasm and the nucleolus (Fig. 3). We asked whether a shift towards more nucleolar protein-like properties [i.e. a greater proportion of charged, particularly positively charged (basic), amino acids] could confer a nucleolar localisation (Fig. 5).

Fusion of mNG to short positively charged (KKKKKKKKKK, RRRRRRRRRR or RKRKRKRKR) sequences conferred a clear nucleolar enrichment (Fig. 5B). These cell lines had high total green fluorescent signal, similar to mNG expressed from the same locus, indicating the charged sequence did not cause protein degradation (Fig. 5D) and, although much protein remained in the cytoplasm, of the protein in the nucleus there was a high nucleolus/nucleoplasm partition (Fig. 5B,E,F). In contrast, fusion with a neutral (GGGGGGGGGG) sequence did not (Fig. 5B,E,F). Fusion with negatively charged (DDDDDDDDDD, EEEEEEEEEE, DEDEDEDEDE) gave some nuclear enrichment, particularly for glutamic acid-containing sequences. However, clear nucleolar targeting/nucleoplasmic exclusion did not occur (Fig. 5B,E,F). This is arguably consistent with the positively charged sequence acting as a nucleolar, but not nuclear, targeting sequence. When ‘assisted’ by an NLS, short positively charged sequences still conferred nucleolar enrichment. Fusion of mNG with KKKKKKRSRE, RRRRRRSRE or RKRKRKRSRE in comparison to the NLS KRSRE alone lead to a clear enrichment in the nucleolus (Fig. 5C,E,F).

A distinctive basic motif, RG or RGG degenerate repeats, is associated with nucleolar localisation in various species, notably for FIB1. The *T. brucei* ortholog of FIB1, NOP1, also has an RG-rich N terminal domain. However, *T. brucei* also has three NOP1 paralogs, and one has all but one RG truncated from the N-terminus. Fewer RGs corresponds to significantly weaker partition to the nucleolus (Fig. S4).

Although some nucleolar proteins do have long runs of positively charged amino acids (e.g. Tb927.5.4270 DEAD helicase and Tb927.9.13340 NOP53), we saw that many proteins do not; charged resides tend to be more dispersed. To test whether charge distributed within a protein can also confer nucleolar targeting, we exploited the natural protein targeting systems of cells. The mitochondrion and glycosome (modified peroxisomes) have N- and C-terminal targeting sequences, respectively – tagging by fusing mNG to the N-terminus of many mitochondrial proteins prevents localisation to the mitochondrion, similarly C-terminal tagging can disrupt glycosomal protein localisation (Fig. S5). We analysed all glycosomal (Fig. S5–C) and mitochondrial (Fig. S5D,E) proteins in the TrypTag data set, which gave a cytoplasmic, nuclear or nucleolar mislocalisation when their targeting sequence was disrupted by being tagged at the N- or C-terminus, respectively. Of these 26, ten localised to the nucleolus. Of the 11 proteins with predicted pI>8.5, eight localised to the nucleolus, a strong enrichment (*P*<10^−5^, Chi squared test). Mitochondrial and glycosomal proteins are normally separated with a membrane from the cytoplasm, nucleoplasm or nucleolus, and should have no specific interactions with nuclear proteins, therefore a physicochemical property like pI is plausibly involved.

Finally, we further investigated short strongly negatively charged sequences, motivated by their ambiguous effect when fused to mNG (Fig. 5) and tendency to arise as nucleolar-enriched motifs (although not at statistically significant levels). We identified two proteins (Tb927.10.2310 and Tb927.9.1560) that localised to the nucleolus with a single DE-rich sequence near the C-terminus of the protein (Fig. S6A). Truncation to remove the acidic sequence (SADDDDDDVEIPEIDMED and SEEEEEEEEPSFEETSSDDDD, respectively) did not prevent nucleolar targeting of either gene, and fusing 10 amino acids of these sequences to mNG did not confer a nucleolar localisation. In fact, in both cases, truncation to remove the acidic sequence slightly, although statistically significantly, increased partitioning to the nucleolus (Fig. S6B).

### Functional consequence of targeting

**Fig. 6.**
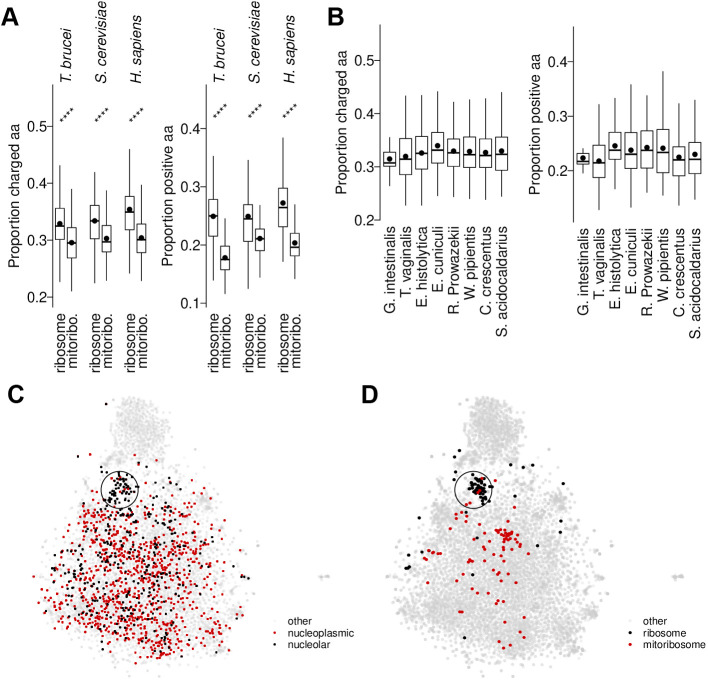
**Many nucleolar proteins have similar amino acid composition to ribosome proteins but not to mitochondrial ribosome proteins.** (A) Proportion of charged (RHKDE) or positively charged (KRH) amino acids found in *T. brucei*, *S. cerevisiae* and *H. sapiens* ribosome and mitochondrial ribosome (mitoribo.) proteins. The box represents the interquartile range and the median is indicated (line), whiskers represent the 5th and 95th percentile, black circles indicate the mean. ns, not significant; *P≤0.05; **P≤0.01; ***P<0.001; ****P≤0.0001 (Wilcoxon signed-rank test). (B) Proportion of charged or positively charged amino acids found in ribosome proteins of organisms which do not have mitochondrial ribosomes, either because they are eukaryotes that have heavily reduced mitochondria (*G.i.*, *T.v.*, *E.h.*, *E.c.*), they are archaea (*R.p.*, *W.p.*) or they are bacteria (alphaproteobacteria, *C.c.*, *S.a.*). Box plots are as described in A. (C) t-SNE plot of all *T. brucei* proteins by amino acid composition (proportion of sequence made up of each residue), highlighting nucleoplasmic and nucleolar proteins. A subset of nucleolar proteins lies in a cluster (circled). (D) The same plot as C, but highlighting ribosome and mitoribosome proteins. aa, amino acids.

Owing to its function in ribosome assembly, the largest flux of protein into the nucleolus is likely ribosome proteins into the granular compartment. However, cells face a challenge – most eukaryotes, including *T. brucei*, assemble at least two types of ribosomes, the mitochondrial ribosome (mitoribosome) in addition to the cytoplasmic ribosome (cytoribosome). We asked whether the mitoribosome proteins had fewer positively charged residues. In humans, yeast and *T. brucei*, cytoribosome proteins have more positively charged residues than mitoribosome proteins (Fig. 6A). The same result was obtained with the first 30 amino acids trimmed from the mitoribosome protein N-terminus, confirming that presence of an N-terminal mitochondrial targeting signal is not responsible. A high proportion of positively charged residues was also seen in the ribosomes of eukaryotes with greatly reduced mitochondria lacking mitoribosomes, archaea ribosomes (the closest prokaryote relative of the eukaryote cytoribosome) and alphaproteobacter (the closest prokaryote relative of the eukaryote mitoribosome) (Fig. 6B), consistent with the low proportion of positively charged residues in mitoribosome proteins being an adaptation to reduce partition of the nucleolus. The distinct amino acid composition of cytoribosome and mitoribosome proteins can be visualised by t-distributed stochastic neighbour embedding (t-SNE) of the proportion of each amino acid in the sequence. A subset of nucleolar proteins had similar overall amino acid compositions (Fig. 6C), and most cytoribosome proteins fell in this cluster, whereas mitoribosome proteins clustered elsewhere (Fig. 6D).

## DISCUSSION

The nucleus is an ancient organelle and, as expected, much of its molecular cell biology is conserved across all eukaryotes. As an early-branching species, *T. brucei* are informative for identifying these ancestral features. Of the nuclear import machinery, many components are conserved (although with significant adaptations) (Canela-Pérez et al., 2019; Keminer and Peters, 1999; Mattaj and Englmeier, 1998; Obado et al., 2016), as is the monopartite KRXR NLS (Marchetti et al., 2000). Our analysis, using genome-wide protein localisation data to quantify nucleolar enrichment, indicated basicity from both short basic IDRs or basic residues throughout the protein, and therefore protein charge, is the conserved feature key for nucleolar targeting.

Our *de novo* search for linear motifs readily re-revealed the KRXR NLS (Figs 2 and 3) but did not reveal a NoLS motif. It instead pointed to the importance of the number of basic residues. Our equivalent analysis of existing genome-wide protein localisation data in humans and yeast (Fig. S3) also showed this pattern. Short linear (poly-R and poly-K) and mixed (poly-RK) sequences were sufficient for nucleolar targeting in *T. brucei* (Fig. 5). This is very similar to results from previous analysis in mammalian cells (Martin et al., 2015; Musinova et al., 2015). We also showed that proteins with dispersed positive charge tend to mislocalise to the nucleolus when their normal targeting sequences are disrupted (Fig. S5). This indicates net charge, rather than a linear motif, is sufficient for nucleolar targeting, and points to basicity being the conserved feature across divergent eukaryotes for nucleolar targeting. However, positive charge alone is a very general feature and is poorly predictive. The nucleolus is also a complex structure, and this does not address targeting to known nucleolar subcompartments.

Although charge alone appeared sufficient for some nucleolar targeting, *T. brucei* nucleolar proteins, as in humans (Stenström et al., 2020), tended to have larger IDRs. The basic charge and low hydrophobicity typical among *T. brucei* nucleolar proteins are associated with condensate formation and IDRs (Quiroz and Chilkoti, 2015; Uversky, 2002). The Das–Pappu diagram of states for polyampholytic IDRs (Das et al., 2015; Holehouse et al., 2017) showed that *T. brucei* nucleolar proteins are often predicted to be polyampholytic coils and hairpins (Fig. S2C). Comparable polymer physics systems indicate this can be favourable for phase separation (Bianchi et al., 2020; Srivastava and Muthukumar, 1996), speaking to the wider question of how the *T. brucei* nucleolus forms.

Nucleolar assembly carries additional importance in *T. brucei* as, in mammalian infective life cycle stages, they form a second distinct RNA Pol I nuclear compartment called the ESB (Navarro and Gull, 2001). The ESB is vital for antigenic variation. It has no function in rRNA transcription or ribosome assembly but does share some components with the nucleolus (RNA Pol I and basal transcription factors) (Nguyen et al., 2012, 2014) and has some unique components (López-Escobar et al., 2022; Faria et al., 2019). Its concurrent existence with the nucleolus means a distinction to their sorting mechanisms exists. How proteins are sorted to the ESB versus nucleolus is therefore an important question for the future.

Protein charge being responsible for nucleolar partition is consistent with LLPS models for nucleolus formation. Some metazoan nucleolar proteins, notably FIB1 and NPM1, can phase separate *in vitro* and form mutually immiscible condensates that mimic nucleolar compartments (Bianchi et al., 2020; Haynes et al., 2006). NPM1 is a major component of the granular compartment in metazoan, with a series of negatively charged acidic tracts in its IDRs. Proteins with characterised basic tracts, which act as NoLSs, including APE1 and ARF, have recently been proposed to partition to the nucleolus through the interaction of their basic arginine motifs with the acidic tracts of NPM1 (Lindström and Zhang, 2006; Lirussi et al., 2012; López et al., 2020; Mitrea et al., 2016, 2018). Based on our evidence in *T. brucei*, we argue that charge interactions with the granular component phase is the general nucleolar targeting phenomenon across eukaryotes.

*T. brucei* nucleolar architecture is incompletely described, but, like most eukaryotes, includes granular (ribosome assembly) and Pol I (transcription) compartments (Daniels et al., 2010). *T. brucei* does not have a clear ortholog of NPM1, although multiple nucleolar proteins with similar acidic tracts and a high proportion of charged amino acids are present. It does, however, have multiple orthologs of FIB1 (called NOP1), which also contain multiple RG motifs that contribute to partitioning to the nucleolus (Fig. S4). This is strongly associated with LLPS, as shown by LAF-1 (*C. elegans* P granules) and DDX4, which have RG motifs necessary for phase separation (Elbaum-Garfinkle et al., 2015; Nott et al., 2015). However, tentatively, NOP1 localises to smaller nucleolar subdomains, and we suspect our analysis is dominated by partition to the larger granular compartment, therefore relating most strongly to ribosome assembly.

We identified a peculiar feature of mitoribosome proteins, that they have a lower proportion of basic amino acids than cytoplasmic ribosome proteins, despite basic amino acids often being common in nucleic acid-interacting proteins and proteins in ribonuclear complexes. *T. brucei* mitoribosomes have an unusually protein-rich composition (Ramrath et al., 2018); however, a low proportion of basic amino acids is not a peculiarity of *T. brucei*, and is also the case in humans and yeast (Fig. 6). To the best of our knowledge, this has not previously been noted and suggests a selection pressure for mitoribosome proteins to be less basic. Evolution of the mitoribosome is complex, having undergone extensive remodelling during its evolutionary course (Ku et al., 2015) after acquisition of the mitochondrion by endosymbiosis of an α-proteobacteria by an ancestral eukaryote (Gray, 2017). This includes acquiring N-terminal mitochondrial localisation signals known as presequences. These presequences are known to be positively charged (Dudek et al., 2013), and despite this, the overall proportion of positively charged residues in mitoribosome protein is still​ lower than in cytoribosome proteins, further implicating a selection pressure. Given that mitochondrial proteins do not generally enter the mitochondrion co-translationally, they necessarily spend some time in the cytoplasm and would have the opportunity to enter the nucleus by accident. The less basic nature of mitoribosome proteins would, therefore, help prevent their partition to the nucleolus. However, we cannot exclude a selection pressure to assist transport across the double mitochondrial membranes.

We saw that many basic mitochondrial proteins can mislocalise to the nucleolus when the mitochondrial localisation signal is disrupted by N-terminal tagging (Fig. S5). Although this indicates that mitochondrial targeting is sufficient to overcome nucleolar targeting arising from protein physicochemical properties, mitoribosome protein targeting would certainly be aided by a mechanism that redirects proteins away from the nucleolus. It might also prevent interference of mitoribosome proteins with cytoribosome assembly in the nucleolus.

In conclusion, proteins with a large number of basic residues, low hydrophobicity and high intrinsic disorder are common among *T. brucei* nucleolar proteins, with basic tracts or an overall basic nature sufficient for nucleolar targeting. Together, this is consistent with LLPS models for nucleolar formation and partitioning of proteins to the compartment. As *T. brucei* is an early-branching eukaryote, and similar features have been implicated in nucleolar targeting in other organisms, this mechanism of nucleolar targeting is likely conserved across eukaryotes. As mitoribosome proteins have a more acidic sequence than cytoribosome proteins, this contributes to cytoribosome versus mitoribosome protein sorting.

## MATERIALS AND METHODS

### Cells and cell culture

Procyclic form *Trypanosoma brucei brucei* strain TREU927 were used, as they were used for the original *T. brucei* (Berriman et al., 2005) genome and the TrypTag genome-wide protein localisation project (Dean et al., 2017). Their identity was recently confirmed by whole genome sequencing. They were grown in SDM-79 (Brun and Schönenberger, 1979) at 28°C, and maintained between ∼6×10^5^ and 2×10^7^ cells/ml by regular subculture.

### Genetic modification

Cell lines stably expressing proteins tagged at the N- or C-terminus with the fluorescent protein mNeonGreen (mNG) (Shaner et al., 2013) were generated by modification of one of the endogenous alleles. Tagging was carried out as previously described, using long primer PCR using the plasmid pPOT v4 BLAST mNG as the template to generate tagging constructs. The template plasmid provides a standard fluorescent protein and drug selection marker coding sequences, and forward and reverse long primers introduce gene-specific 80 bp 5′ and 3′ homology arms – to either the 5′ UTR and start of the target gene ORF or the end of the target gene ORF (excluding the stop codon) and the 3′ UTR, for N- and C-terminal tagging, respectively (Dean et al., 2015) (primer sequences in Table S1). High-throughput electroporation was used to transfect *T. brucei* with the tagging, constructs, which integrate into the target locus by homologous recombination (Dyer et al., 2016). 10 µg/ml blasticidin S hydrochloride (Melford, B12150) was used to select for successful transfectants.

Cell lines stably expressing truncated tagged proteins were generated as for tagging except with shifted base matching within the target gene ORF to introduce a truncation at the tagged terminus, as previously described (Dean et al., 2015). For N-terminal truncation, base matching to the target gene ORF in the reverse primer was shifted the necessary number of codons into the start of the ORF and for C-terminal truncation base matching to the target gene ORF in the forward primer was shifted the necessary number of codons into the end of the ORF (primer sequences in Table S1).

Cell lines expressing mNG with a N- or C-terminal candidate targeting sequence were also generated using a similar PCR-based method. Here, the homology arms were designed such that one copy of PFR2 in the multi-copy PFR2 array is replaced by the mNG and drug selection marker coding sequences. Using the standard pPOT primer binding sites (Dean et al., 2015), a candidate targeting sequence of up to 10 codons can be fused to the mNG coding sequence, using 50 bp from the target site (for homologous recombination) and 30 bp encoding the targeting sequence on the forward or reverse primer for introduction to the C- or N-terminus of mNG, respectively (primer sequences in Table S1).

### Microscopy

Live-cells were stained with Hoechst 33342 (Sigma-Aldrich, B2261) and adhered, live, to glass slides as previously described (Dean and Sunter, 2020). mNG and Hoechst 33342 fluorescence and phase-contrast micrographs were captured on the same microscope and using identical settings as the TrypTag genome wide protein localisation project, a DM5500 B (Leica) upright widefield epifluorescence microscope using a plan apo NA/1.4 63× phase contrast oil immersion objective (Leica, 15506351) and a Neo v5.5 (Andor) sCMOS camera using MicroManager (Edelstein et al., 2010).

### Automated image analysis

Image analysis builds on our previous approaches (Wheeler, 2020; Wheeler et al., 2012) using ImageJ (Collins, 2007). All images analysed were at 0.103 μm/px. They were first flat-field corrected by subtracting the median of all images captured on a particular day. To identify cells, phase-contrast images were pre-processed by sequential Gaussian unsharp filters with radii from 1 to 35 px at 5 px steps with 0.4 weight, then an intensity threshold of the image mean minus 1× s.d. was applied. Cells were taken as objects between 2000 and 7000 px^2^, with a minimum pixel value at least two s.d. under the mean (Fig. 1A).

To identify nuclei, Hoechst 33342 fluorescence images were pre-processed with a 1 px radius Gaussian blur and a rolling ball subtraction with radius 15 px. Local maxima with prominence over 1.5× image s.d. were taken as DNA-containing objects, with a threshold equal to 0.4× the local maxima (Fig. 1A). *T. brucei* have two DNA-containing structures, the nucleus (N) and kinetoplast (K) and the kinetoplast divides before the nucleus in the cell cycle; therefore, in cells with two DNA-containing structures (expected to be 1K1N) the largest was the taken as the nucleus, in three structure cells (expected to be 2K1N) the largest was taken as a nucleus and four structure cells (expected to be 2K2N) the largest two were taken as nuclei. The mean nucleus radius *r* was taken as the average of the major and minor axes of an ellipse fitted to the thresholded object. Nucleoli appeared as small circular regions of lower Hoechst 33342 in the nucleus. To identify nucleoli, the darkest point within the nucleus at least *r*/8 from its edges was taken as the nucleolar centre and assumed to have a radius *r*/4 (Fig. 1B).

Nuclear/cytoplasm partition was taken as the ratio of mean nuclear signal to mean cytoplasmic signal in the mNG fluorescence channel, and nucleolar/nucleus partition was taken as the ratio of mean nucleolar signal to mean nucleoplasm (i.e. excluding the nucleolus) signal (Fig. 1C). Data for the TrypTag genome tagging project dataset represent the mean partition for all cells (typically >200), plotting N- and C-terminally tagged cell lines separately. Other data was further filtered to exclude cells not expressing the fluorescently tagged protein. On average, 360 cells were analysed per cell line; all plots show data from at least 20 cells.

### Cell line validation

Protein samples from a subset of cell lines were subject to western blotting to confirm expression of an mNG fusion protein of the expected molecular mass. The primary antibody was monoclonal anti-mNeonGreen (Chromotek 32F6), diluted 1:100. The secondary antibody was anti-mouse-IgG conjugated to peroxidase (Jackson ImmunoResearch, 715-035-150), diluted 1:10,000.

Correct genetic modification was confirmed by sequencing across the site of expected genetic modification (primer sequences in Table S2). For endogenously tagged cell lines and truncations, PCR using gDNA template and a primer pair in the target gene ORF and mNG (forward and reverse, respectively, for C-terminal tagging, reverse and forward for N-terminal tagging) was used to amplify part of the modified locus. For mNG fused to an NLS, a primer pair in mNG and the UTR of the target locus (3′ for NLS::mNG fusions, 5′ for mNG::NLS fusions) was used. Sanger sequencing of the PCR product confirmed the expected modifications.

In all cell lines, the protein of interest was fused to mNG allowing assessment of approximate expression level from light microscopy. Cell lines with anomalously weak expression, perhaps arising from mis-integration of the tagging construct, misfolding of the mNG or degradation of an unstable fusion product, were excluded.

To confirm that observed localisations were repeatable, up to three independent attempts (using newly synthesised primers) were made at generating each non-clonal cell line. If all attempts at generating a cell line gave a visually similar appearance, then one was selected for quantitation. The number of successful attempts for generating each cell line is listed in Table S1.

### Protein primary sequence analysis

Meme (Bailey et al., 2009) version 5.1.1 was used to identify linear motifs enriched in nuclear and nucleolar proteins, searching for motifs with one occurrence per sequence and widths between 4 and 16. IDRs were identified using IUPred2A (Erdős and Dosztányi, 2020; Mészáros et al., 2018), taking residues with a score over 0.5 as disordered.

Human protein localisations were taken from the Human Cell Atlas (accessed Dec 2020), taking any proteins annotated with terms including ‘nucleoli’ as nucleolar, and proteins annotated with any nuclear lumen structures as a nuclear (Thul et al., 2017). Yeast localisations were taken from the Yeast GFP Fusion Localization Database (accessed Dec 2020), using their nucleolar and nuclear annotations (Huh et al., 2003).

*T. brucei* protein sequences were taken from TriTrypDB v51 (Aslett et al., 2010); for all other species, protein sequences were taken from UniProt. *T. brucei* cytoribosome and mitoribosome protein lists were derived from those identified by affinity purification and/or cryoelectron microscopy structures (Hashem et al., 2013; Saurer et al., 2019; Zíková et al., 2008; Ramrath et al., 2018). In other species, lists were derived from UniProt protein annotations – for example ‘60S ribosomal protein LX’ or ‘40S ribosomal protein SX’ for human cytoribosomes.

### Data plotting and statistics

Unless otherwise indicated, box plots show the median and interquartile range, whiskers represent the 5th and 95th percentile, and black circles indicate the mean. Statistical significance was assessed using the Wilcoxon signed-rank test, and is presented as ns when not significant, *P≤0.05, **P≤0.01, ***P≤0.001, ****P≤0.0001.

## Supplementary Material

10.1242/joces.259701_sup1 Supplementary information
