# Supplementary material for: Nucleolar targeting in an early-branching eukaryote suggests a general mechanism for ribosome protein sorting
Source: J Cell Sci. 2022 Oct 4;135(19):jcs259701. doi: 10.1242/jcs.259701 (PMC9659390; doi:10.1242/jcs.259701)
Supplement: Supplementary information [file joces-135-259701-s1.pdf]

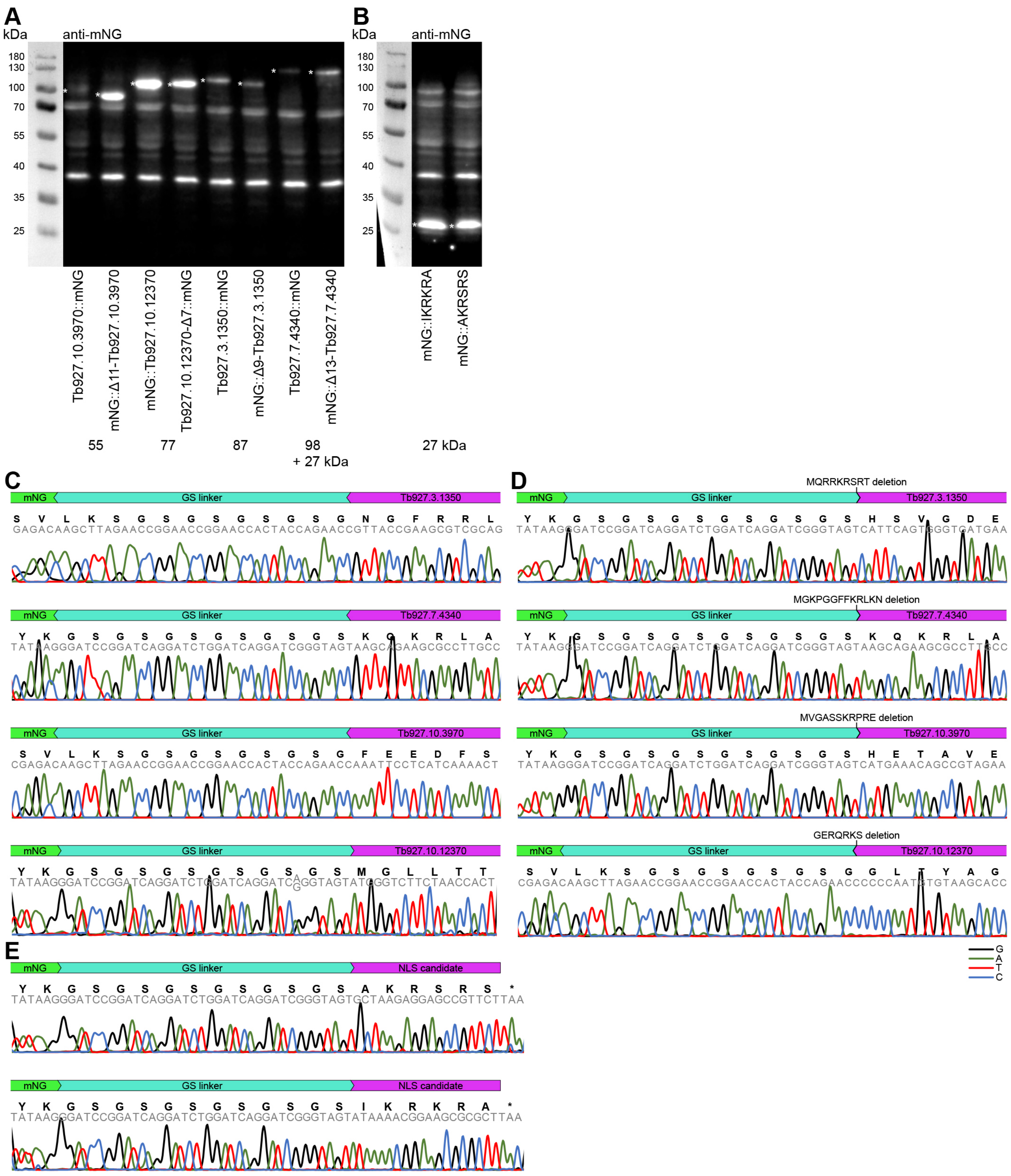


**Fig. S1. Validation of the tagging, truncation and fluorescent protein-targeting sequence fusion methodologies.**

A. Anti-mNG Western blot of whole cell lysates of representative cell lines expressing tagged and truncated nuclear proteins from Figure 3B,C. Approximate expected molecular weight (unmodified protein, without the ~28 kDa mNG tag) is shown at the bottom. Truncation is expected to remove ~0.1 kDa per amino acid. Major bands specific to the cell lines are marked with asterisks.

Journal of Cell Science • Supplementary information

B. Anti-mNG Western blot of whole cell lysates of representative cell lines expressing mNG-NLS fusions from Figure 3B,C. Approximate expected molecular weight is ~28 kDa, NLS sequences are ~0.7 kDa. Major bands specific to the cell lines are marked with asterisks.
C. Electropherograms from sequencing confirmation of expected mNG ORF fusion to the tagged gene ORF, for the tagged cell lines shown in A. PCR products spanning the site of mNG integration in the genome were Sanger sequenced using an upstream reverse (for N terminal) or downstream forward mNG primer.
D. As for C, but for the cell lines with truncations removing a candidate NLS from A.
E. As for C, but for the cell lines expressing mNG fused to a candidate NLS from B.

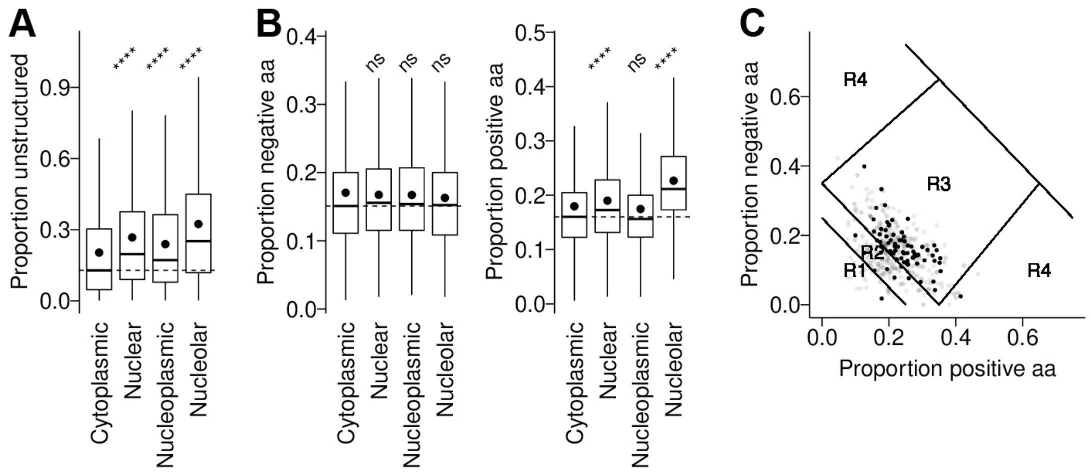


**Fig. S2. Unstructured domains in *T. brucei* nucleolar proteins.**

A. Proportion of predicted intrinsically disordered (IUPRED) residues in cytoplasmic, nuclear, nucleoplasmic or nucleolar proteins, as classified by the cutoffs indicated in Figure 2.

B. Proportion of positive (RHK) or negative (DE) charged amino acids found in the predicted unstructured domains of nuclear, nucleoplasmic or nucleolar proteins.

Boxes represent median and quartile range, whiskers represent the 5th and 95th percentile, points indicate the mean. Statistical significance was assessed using the Wilcoxon signed-rank test (ns not significant, * $p \leq 0.05$, ** $p \leq 0.01$, *** $p \leq 0.001$, **** $p \leq 0.0001$).

C. Diagram of states classification of predicted intrinsically disordered nucleolar protein domains. Strongly nucleolar (nucleolus/nucleus partition >1.2) are plotted in black. Specific regions are indicated: R1 globules, R2 globule and coil chimaeras, R3 polyampholytic coils or hairpins, R4 polyelectrolytic semi-flexible rods or coils.

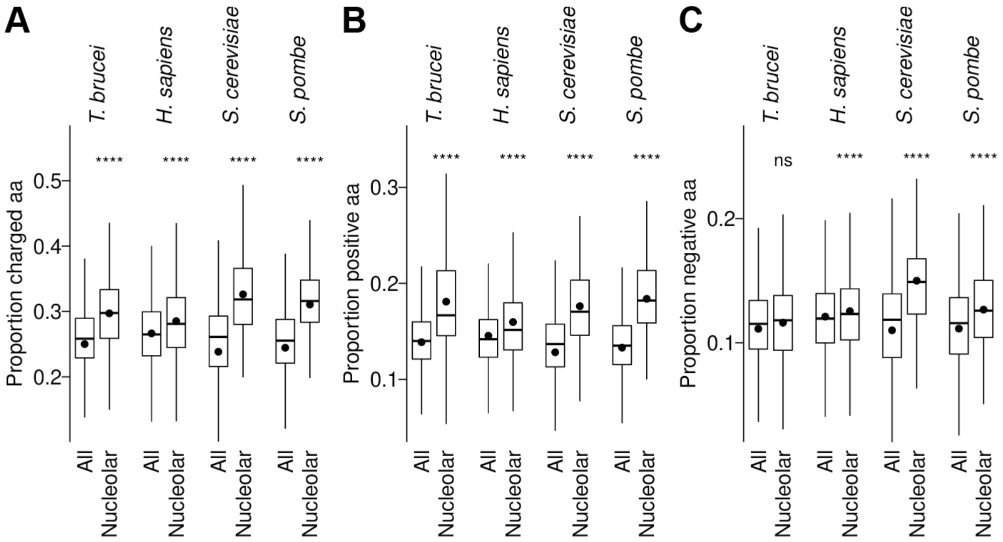


**Fig. S3.** ***T. brucei,*** **yeast and human nucleolar proteins tend to have many positively charged amino acids.**

A. Proportion of charged (RHKDE) amino acids found in proteins annotated as nucleolar in T. brucei, in comparison an equivalent analysis of all proteins encoded in the genome from the *S. cerevisiae*, *S. pombe* and *H. sapiens* genome-wide protein localisation projects.

B. As for A, but for positively charged amino acids (RHK).

C. As for A, but for negatively charged amino acids (DE).

Boxes represent median and quartile range, whiskers represent the 5th and 95th percentile, points indicate the mean. Statistical significance was assessed using the Wilcoxon signed-rank test (ns not significant, * p≤0.05, ** p≤0.01, *** p≤0.001, **** p≤0.0001).

Journal of Cell Science • Supplementary information

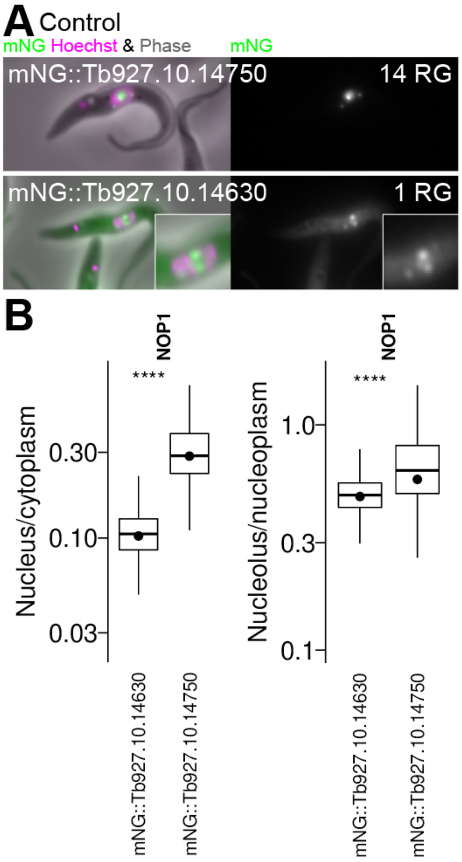


**Fig. S4. RGGs in nucleolar scaffolds are required for their partition to the nucleolus.**

A. Localisation of two paralogs of T. brucei NOP1, one with 14 RGs in the sequence and one with an N terminal truncation leaving one RG.

B. Plots of automated quantitation of the nucleus/cytoplasm and nucleolus/nucleoplasm mNG fluorescence signal partition from the cell lines in A.

Boxes represent median and quartile range, whiskers represent the 5th and 95th percentile, points indicate the mean. Statistical significance was assessed using the Wilcoxon signed-rank test (ns not significant, * p≤0.05, ** p≤0.01, *** p≤0.001, **** p≤0.0001).

Journal of Cell Science • Supplementary information

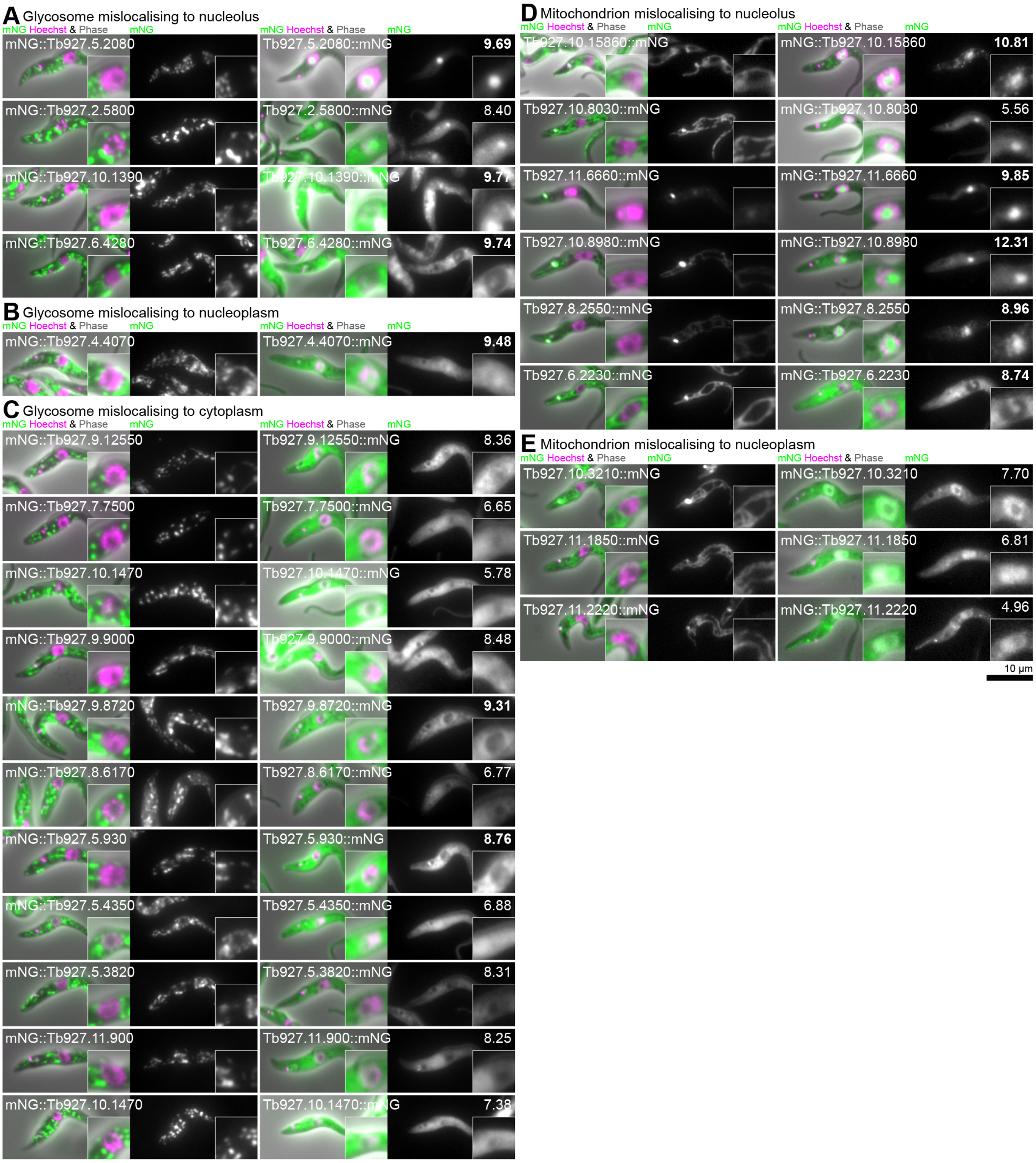


**Fig. S5. Basic proteins tend to mislocalise to the nucleolus when normal targeting sequences are disrupted.**

A-C. Localisation of glycosomal proteins (with a C terminal glycosome/peroxisome targeting sequence) which mislocalise to the cytoplasm, nucleoplasm and/or nucleolus when tagged on the N terminus.

A. Glycosomal proteins which mislocalise to the nucleolus when tagged on the C terminus.

B. Glycosomal proteins which mislocalise to the nucleoplasm when tagged on the C terminus.

C. Glycosomal proteins which mislocalise to the cytoplasm when tagged on the C terminus.

Journal of Cell Science • Supplementary information

D-E. Localisation of all mitochondrial proteins (with an N terminal mitochondrial targeting sequence) which mislocalise to the cytoplasm, nucleoplasm and/or nucleolus when tagged on the N terminus. D. Mitochondrial proteins which mislocalise to the nucleolus when tagged on the N terminus.

E. Mitochondrial proteins which mislocalise to the nucleoplasm when tagged on the N terminus. Nomitochondrial proteins mislocalise to the cytoplasm. For each cell line, the mNG fusion protein pI is shown in the top right, in bold if >8.50.

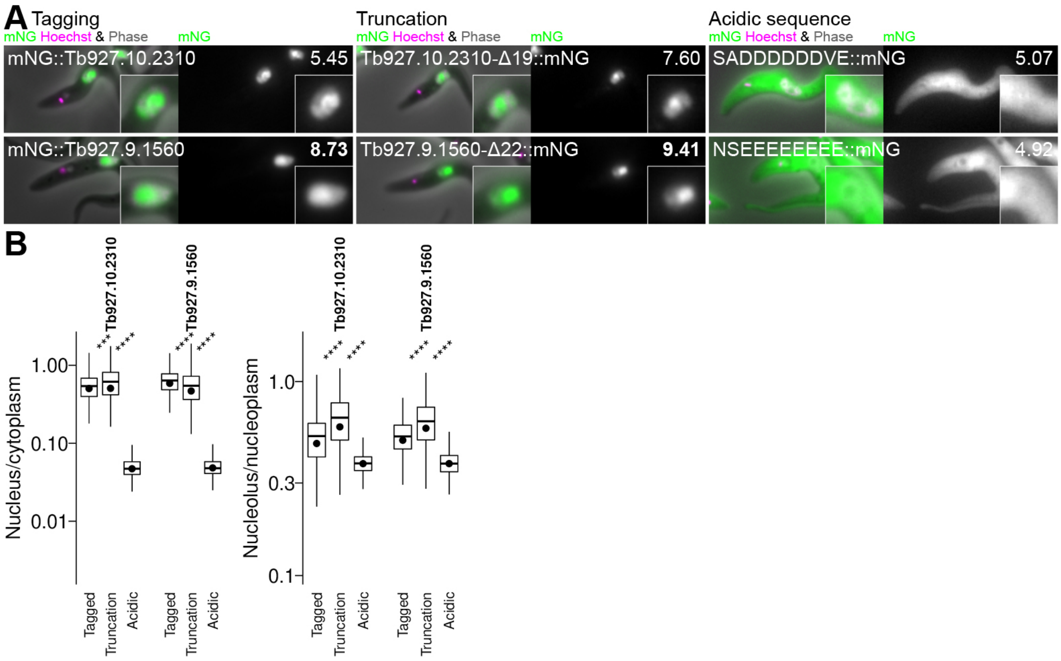


**Fig. S6. Acidic sequences in nuclear proteins are not required for nucleolar targeting.**

A. Testing acidic runs for roles in nucleolar sequencing for 2 nuclear proteins with a single acidic run near the C terminus. Localisation of the protein by tagging at the endogenous locus, localisation following truncation to remove the C terminal acidic run and replacement with mNG and localisation of mNG fused to 10 amino acids of the acidic run. For each cell line, the number mNG fusion protein pI is shown in the top right, in bold if >8.50.
B. Plots of automated quantitation of the nucleus/cytoplasm and nucleolus/nucleoplasm mNG fluorescence signal partition from the cell lines in A.
Boxes represent median and quartile range, whiskers represent the 5th and 95th percentile, points indicate the mean. Statistical significance was assessed using the Wilcoxon signed-rank test (ns not significant, * p≤0.05, ** p≤0.01, *** p≤0.001, **** p≤0.0001).

**Table S1.** Primers used for cell line generation and number of replicates generating cell lines.

Click here to download Table S1

**Table S2.** Primers used for confirmation of genetic modification by PCF from genomic DNA then sequencing.

Click here to download Table S2
