# Supplementary material for: Nucleolar targeting in an early-branching eukaryote suggests a general mechanism for ribosome protein sorting
Source: J Cell Sci. 2022 Oct 4;135(19):jcs259701. doi: 10.1242/jcs.259701 (PMC9659390; doi:10.1242/jcs.259701)
Supplement: Supplementary file 1 [file joces-135-259701_review_history.pdf]

Nucleolar targeting in an early-branching eukaryote suggests a general mechanism for ribosome protein sorting

Milad Jeilani, Karen Billington, Jack Daniel Sunter, Samuel Dean and Richard John Wheeler
DOI: 10.1242/jcs.259701

Editor: Maria Carmo-Fonseca

Review timeline
Original submission: 18 December 2021
Editorial decision: 24 January 2022
First revision received: 24 August 2022
Accepted: 24 August 2022

Original submission

First decision letter

MS ID#: JOCES/2021/259701

MS TITLE: Nucleolar targeting in an early-branching eukaryote suggests a general physicochemical mechanism for ribosome protein sorting

AUTHORS: Milad Jeilani, Karen Billington, Jack Daniel Sunter, Samuel Dean, and Richard John Wheeler
ARTICLE TYPE: Research Article

We have now reached a decision on the above manuscript.

To see the reviewers' reports and a copy of this decision letter, please go to: https://submit-jcs.biologists.org and click on the 'Manuscripts with Decisions' queue in the Author Area. (Corresponding author only has access to reviews.)

As you will see from their reports, the reviewers' recommendations regarding publication are somewhat mixed. While referee #2 considers that your study represents too little progress for warranting publication, the other two thought that the work was potentially quite interesting and significant but all also raised a number of concerns that must be dealt with. Please address the issues raised by the three reviewers as thoroughly as possible. If you think that you can deal satisfactorily with the criticisms on revision, I would be pleased to see a revised manuscript. I would then return it to the reviewers.

We are aware that you may be experiencing disruption to the normal running of your lab that makes experimental revisions challenging. If it would be helpful, we encourage you to contact us to discuss your revision in greater detail. Please send us a point-by-point response indicating where you are able to address concerns raised (either experimentally or by changes to the text) and where you will not be able to do so within the normal timeframe of a revision. We will then provide further guidance. Please also note that we are happy to extend revision timeframes as necessary.

Please ensure that you clearly highlight all changes made in the revised manuscript. Please avoid using 'Tracked changes' in Word files as these are lost in PDF conversion.

© 2022. Published by The Company of Biologists under the terms of the Creative Commons Attribution License (https://creativecommons.org/licenses/by/4.0/).

I should be grateful if you would also provide a point-by-point response detailing how you have dealt with the points raised by the reviewers in the 'Response to Reviewers' box. Please attend to all of the reviewers' comments. If you do not agree with any of their criticisms or suggestions please explain clearly why this is so.

Reviewer 1

Advance summary and potential significance to field

The manuscript submitted by Jeilani et al describes a key feature responsible for protein targeting to the nucleolus in T. brucei. The authors started by exploiting available data in their TrypTag database (Dean et al 2017) to identify proteins that localise mostly (but not exclusively in some cases) to the nucleus and the nucleolus. The identification of a canonical NLS validated the approach. By truncating a number of protein candidates and fusing mNG with sequences of interest, the authors then demonstrated that basic amino acids (both in short IDRs and distributed through a protein) play a major role in nucleolar targeting.
These findings are consistent with an LLPS model, which has been proposed for nucleolar assembly in other eukaryotes and suggest that the mechanism of nucleolar targeting is likely to be conserved across eukaryotes.
Interestingly, and as noted by the authors, in the mammalian-infective stage, T. brucei forms a second distinct Pol-I compartment, and how proteins are differentially sorted between the ESB vs the nucleolus remains enigmatic and an important question for the future.

Comments for the author

The work is a nice piece of cell biology, and no additional experiments appear to be required, as the conclusions are supported by the data.
However, there are a few aspects that would benefit from further clarification/discussion:
-It would be helpful to include an image illustrating the nuclear and nucleolar segmentation using the automated high content image analysis.
-Regarding the proteins that have a nuclear and/or nucleolar localisation as well as some cytosolic signal, it would be important to comment on how that might have biological significance (proteins might have dual localisation and possibly even shuttle between the two compartments) or might be artificially created by tagging (e.g. disruption of UTR-mediated control therefore affecting protein endogenous levels; partial disruption of nuclear import both through nuclear importins and/or piggybacking; etc).
-When truncating KRXR motifs in the 8 candidate genes, 1 retained complete nuclear localisation and 5 out of 7 partially retained nuclear localisation. Can you elaborate on why do you think this is?
-It is important to provide data (PCR, sequencing and/or western-blot, etc) showing that the truncations and fusions in Figs. 3 and 5 were correctly generated (at least for a few genes just to show that such validation was performed).
-The authors state that “The canonical KRXR NLS is strongly enriched in nucleolar and nucleoplasmic genes – found in ~50% – however presence of KRXR alone is not a good predictor of a nuclear localisation.”
What is the % of proteins that have a KRXR motif and do not localise to the nucleus?
-It could be useful to the wider community to add a line naming specific KRXR sequences and respective flanking amino acids that can be used to successfully target proteins to the nucleus in T. brucei.
Also, minor changes to the figures and text would improve their accessibility to the general reader:
-Fig. 1A lacks a scale bar. There is also an overlap between text in 1A and 1B. In both 1B and 1C the axis are missing and the labels are imperceptible (making both the symbols and the font bigger would be helpful).
The legend in Fig. 1 refers to Fig. 2, which is a bit odd, the other way around would be more acceptable. This has to do with the fact that panels 1B and 1C are the same as in 2A and 2C, respectively, thus a possibility would be to merge Figure 1 and 2, where 2A-D would be followed by 1A and then 2E.
-Fig. 3A-C panels lack a scale bar

© 2022. Published by The Company of Biologists under the terms of the Creative Commons Attribution License (https://creativecommons.org/licenses/by/4.0/).

-Figs. 3 and 5: the legend should contain information on the number of clones and/or independent experiments that were performed, as well the average number of cells used in each condition. Of for simplicity, that information can be included in the corresponding section in M&M.
-Fig. 5: the “E” label overlaps with the axis label
-Fig. 6C-D: some of the darker grey dots are difficult to visualise, a different colour scheme would be helpful.
-The supplementary figures need to be reorganised as in the text, they are not mentioned in order (they are mentioned as follows: S1 / S5 / S3 / S4 and there is no mention of Fig. S2).

Reviewer 2

Advance summary and potential significance to field

This MS is a submission from a group of ex-Gull lab PIs and others, and builds upon the proteome-wide localization studies. I was genuinely looking forward to reading this work as insights into the biology of trypanosomes arising from the tagging project would be of considerable interest, as would some advances in understanding organelle biogenesis. Sadly what we have here, as far as I can understand, is a poorly presented set of experiments that in many places are not well thought out, badly written and lack of obvious argumentation. The overall contribution here is limited, and I recommend reject. There seems here to be a failure to engage, with the literatire, the biology or attempts to work with the reader for clear exposition.

Awkward 1st line and sense of final line in abstract is incomprehensible.

Page numbers on an MS are essential.

Nucleolus size? Well - by what criteria - assume volume as ER is probably targets thing in terms of area although that is membrane bound I suppose. So what anyway? Size matters…

noitpecxe on si iecurb amosonapyrT etisarap ralullecinu ehT What does this sentence mean exactly? Apologies, but cut and paste rendered this a mirror image…
I am not really convinced that there is evidence as cited that the nucleolus is distinct between life stages.
This seems a bit weak to me as the procyclin and ESBs are both sensu stricto extra nucleolar. I don’t think this is needed as this is likely the most divergent from canonical organisms that such an analysis has been performed.
There are many NLSs, of distinct structures and forms, and a better ref would be DOI: 10.1007/s00894-017-3420-y Not ALL eukaryotes, but would support MANY or WIDESPREAD F1 Resolution in B and C is poor. Also - color the different cohorts in the scatterplots?
F2 Panel B the scale here needs to be linear. I cannot work out where the 30kDa would sit.
Dashed lines - in some cases we are referred to one line or even none, but in all of these scattergrams there are two!
It may help if one or two known nucleolar/nucleolar proteans were annotated in these plots.
Panel E - what is the X-axis? Assume residue number but from what?
F3 This is just a mess - data are likely pretty good but arrangement, annotation etcetera leaves a lot to be desired and also makes the thing hard to understand. There are no axes on panel E and F or indicators of what the whiskers, boxes and dots indicate. This then becomes difficult to evaluate as I am left to assume what these data mean rather than being confident. A reviewer should not have to work herself so hard.
F4 - Does there not need to be a total cell category here? Also, I think work using LopIT and similar has identified nucleolar cohorts in several additional species - Arabidopsis certainly comes to mind and as the current taxon sampling is Opistokhonta versus a kinetoplastid, not great. This latter could presumably be improved by chosing one or two additional Kinetoplastids and analysing the relevant paralogs?
I’m not very impressed with the KKKKK etcetera and RRRR etcetera strategy. As the authors state, a lot of these proteins remain in the cytosol, and a huge issue here is if these are folded, generically sticky etcetera.
I’m also unclear as to if westerns have been performed here to ensure the constructs are intact and not partially degraded. It is also possible that these basic proteins are simply binding to a carrier

© 2022. Published by The Company of Biologists under the terms of the Creative Commons Attribution License (https://creativecommons.org/licenses/by/4.0/).

that recruits to the nucleus for example. This is a very crude approach. The comment that the NLS improves nucleolar targeting of these probes is really not very helpful - if you provide an additional mechanism to recruit to the organelle, then perhaps this is obvious.
F6 - This is also well known, and simply showing this demonstrates a lack of understanding the literature.
Interesting that there is no citation of recent work demonstrating the divergence of the kinetoplastid mitoribosome, a spectacular story that deserves recognition here.

Overall the work fails to meet the first criterion for acceptable work for JCS.

Comments for the author

None

Reviewer 3

Advance summary and potential significance to field

This manuscript presents a research work that analyzes nucleolar protein properties from the parasitic protozoa Trypanosoma brucei. The interest of this investigation is to provide additional insight to protein separation into the nucleolus. From an evolutionary point of view, to address the search of nucleolar partition determinants in an early branching organism such as T. brucei is a relevant question to pursue. The selection of proteins for this study was based on the Tryp Tag data base resource that provides cellular location of proteins encoded in the T. brucei genome, tagged and analyzed by microscopy. With this approach, the work presented provides clear evidence to support their conclusion that targeting to the nucleolus is mediated by positive charges in the nucleolar proteins, and that these charged residues aid towards a differential partition of mitochondrial and cytoplasmic ribosomal proteins. The manuscript is suitable for its publication, minor issues are mentioned below.

Comments for the author

Minor issues to be addressed:
1. In legend to figure S1 the indicator B is repeated. The second B should be changed to C.
2. Legend to figure S3: A-C and A. B. C. Legend states that the mis-localized fusion proteins are the ones tagged on the N-terminus. Nevertheless, as shown in the corresponding images, the mis-localized proteins at those tagged on the C- terminus. The legend should be corrected in all 4 cases.
3. A statement regarding the multifunctionality of the nucleolus may be included to improve the manuscript.

First revision

Author response to reviewers' comments

We have comprehensively addressed the reviewers’ comments, see below. Note, that through doing so, the figure numbers have changed. We use the new figure numbering in our responses.

Reviewer 1 Advance Summary and Potential Significance to Field:

The manuscript submitted by Jeilani et al describes a key feature responsible for protein targeting to the nucleolus in T. brucei. The authors started by exploiting available data in their TrypTag database (Dean et al, 2017) to identify proteins that localise mostly (but not exclusively in some cases) to the nucleus and the nucleolus. The identification of a canonical NLS validated the approach. By truncating a number of protein candidates and fusing mNG with sequences of

© 2022. Published by The Company of Biologists under the terms of the Creative Commons Attribution License (https://creativecommons.org/licenses/by/4.0/).

interest, the authors then demonstrated that basic amino acids (both in short IDRs and distributed through a protein) play a major role in nucleolar targeting. These findings are consistent with an LLPS model, which has been proposed for nucleolar assembly in other eukaryotes and suggest that the mechanism of nucleolar targeting is likely to be conserved across eukaryotes.

Interestingly, and as noted by the authors, in the mammalian-infective stage, T. brucei forms a second distinct Pol-I compartment, and how proteins are differentially sorted between the ESB vs the nucleolus remains enigmatic and an important question for the future.

Thank you for the support regarding the interest and rigour of this work, and we have endeavoured to address your detailed comments below.

Reviewer 1 Comments for the Author:

The work is a nice piece of cell biology, and no additional experiments appear to be required, as the conclusions are supported by the data.

However, there are a few aspects that would benefit from further clarification/discussion:

-It would be helpful to include an image illustrating the nuclear and nucleolar segmentation using the automated high content image analysis.

We have added this to Figure 1, illustrating the nucleus and nucleolus segmentation. Hopefully this is an effective illustration of the segmentation process which supports the text description.

-Regarding the proteins that have a nuclear and/or nucleolar localisation as well as some cytosolic signal, it would be important to comment on how that might have biological significance (proteins might have dual localisation and possibly even shuttle between the two compartments) or might be artificially created by tagging (e.g. disruption of UTR-mediated control therefore affecting protein endogenous levels; partial disruption of nuclear import both through nuclear importins and/or piggybacking; etc).

Absolutely, we completely agree that a nuclear localisation is not a binary classification, with significant possibility for stronger or weaker enrichment, very dynamic shuttling processes, etc. This is an area where protein localisation (rather than, for example, a nuclear proteome from mass spectrometry) is advantageous for a quantitative nuclear enrichment analysis. We have added a short consideration of these points to the text.

Regarding the very valid concerns of masking a targeting sequence with a tag, altering expression level and thus nuclear partition, etc. we have already partially addressed this. As many proteins were tagged on both the N and C terminus, we could analyse the correlation of nucleus/cytoplasm partition and nucleolus/nucleus partition (Figure 2D). We have clarified the text here to emphasise that mismatch between N and C terminal tagging may well be artefactual, and potentially a biologically informative artefact.

-When truncating KRXR motifs in the 8 candidate genes, 1 retained complete nuclear localisation and 5 out of 7 partially retained nuclear localisation. Can you elaborate on why do you think this is?

Strong nuclear localisation would presumably be preserved when an alternate targeting mechanism exists, e.g. an additional cryptic/unusual (therefore overlooked) NLS, a binding domain that enables interaction with an abundant nuclear protein, etc. In the case of Tb927.3.1350 it is a very basic protein with a nucleolar localisation, so likely retaining its nucleolar localisation due to this property.

Partial remaining nuclear localisation is likely as simple as a lack of nucleus *exclusion* mechanism, leading to limited nuclear protein attaining its normal localisation while partition is overall significantly disrupted.

We have added a note to the relevant results paragraph to this effect.

© 2022. Published by The Company of Biologists under the terms of the Creative Commons Attribution License (https://creativecommons.org/licenses/by/4.0/).

-It is important to provide data (PCR, sequencing and/or western-blot, etc) showing that the truncations and fusions in Figs. 3 and 5 were correctly generated (at least for a few genes just to show that such validation was performed).

To demonstrate the reliability of the tagging, truncation and mNG-targeting sequence methodologies we selected a random subset of gene tagging/gene truncation pairs and mNG-targeting sequence fusions where we had retained the cell lines. anti-mNG Western blotting and sequencing across the site of the expected modification confirmed correct generation.

We also did not previously make it clear that that almost all cell lines shown have also been generated completely independently at least twice, many three or more times (see below).

-The authors state that "The canonical KRXR NLS is strongly enriched in nucleolar and nucleoplasmic genes – found in ~50% – however presence of KRXR alone is not a good predictor of a nuclear localisation." What is the % of proteins that have a KRXR motif and do not localise to the nucleus?

This is also ~50%: 1604 Tb927 proteins have a KRXR motif, of which 1350 were covered by our localisation data. Of these, 749 non-nuclear proteins had KRXR. According to our analysis, the number of nuclear proteins was 1396 of which 795 had KRXR. We have updated the text with more precise percentages for both. NB. 749 and 795 sum to more than 1350, the excess is the result of treating N and C terminal tagging data independently.

-It could be useful to the wider community to add a line naming specific KRXR sequences and respective flanking amino acids that can be used to successfully target proteins to the nucleus in T. brucei.

The complete set of NLS sequences we tested/validated sequences are those in Figure 3B and 2F. For the general motif of XKRXRX, the most common amino acid in each position across all nuclear proteins was X1: R, X2: R, X3: E, pointing to an optimal NLS sequence of RKRRRE. We have added this to the text.

Combined with your above comment, this inspired a minor analysis which may be of interest. Taking the set of XKRXRX motifs present in nuclear and non-nuclear proteins we can test which amino acids occur disproportionately often in nuclear vs. non-nuclear proteins. This gives is X1: R, X2: P, X3: D/E, pointing to the true optimal (most discriminative) NLS sequence being RKRPR[D/E]. Similarly, some amino acids were clearly not under- represented in nuclear protein XKRXRX: X1: E/V/I/L, X2: L/V/I, X3: L/K. This indicates a least- effective NLS sequence of [E/V/I/L]KR[L/V/I]R[L/K].

Also, minor changes to the figures and text would improve their accessibility to the general reader:

-Fig. 1A lacks a scale bar. There is also an overlap between text in 1A and 1B. In both 1B and 1C the axis are missing and the labels are imperceptible (making both the symbols and the font bigger would be helpful).

We have corrected the text overlap and apologise for the omission of a scale bar – we had taken care to ensure all images are shown at the same magnification for consistency, however had forgotten to include the actual scale!

Regarding the plots in Figure 1E and F, it is necessary to have transparent small points as we are trying to display data from thousands of cell lines with data point labels on top – it is an opaque black mess with higher opacity or larger points. Similarly, fitting the cell line labels necessitates a small font size. Both are well visible in the original files and reproduce with sufficient clarity when printed at A4 size – however we will check at the proofing stage that they are indeed visible.

The legend in Fig. 1 refers to Fig. 2, which is a bit odd, the other way around would be more acceptable. This has to do with the fact that panels 1B and 1C are the same as in 2A and 2C,

© 2022. Published by The Company of Biologists under the terms of the Creative Commons Attribution License (https://creativecommons.org/licenses/by/4.0/).

respectively, thus a possibility would be to merge Figure 2 and 2, where 2A-D would be followed by 1A and then 2E.

Thank you for highlighting this. We have carefully rearranged the text and figures to better reflect the order they are referred to in the text. Combined with your request for a figure describing the image analysis, we opted to combine the previous Figure 1 with this new image analysis Figure.

-Fig. 3A-C panels lack a scale bar

We have added the scale bar.

-Figs. 3 and 5: the legend should contain information on the number of clones and/or independent experiments that were performed, as well the average number of cells used in each condition. Of for simplicity, that information can be included in the corresponding section in M&M.

All measurements shown are from a single cell line; however, as noted above, the majority of cell lines were generated independently at least twice with visually consistent results. As this supports the robustness of the dataset, we have prepared a supplementary table which lists the number of independent times each experiment was replicated (albeit not quantified). We have added this information to the legends and methods.

Through reviewing this data, we were a little unsatisfied with the success rate generating candidate NLS-mNG fusions. We therefore repeated these transfections, successfully obtaining the mNG::AKRSRS and mNG::IKRKRA lines. Both were indeed functional NLSs.

As you suggest, we have also added typical and minimum numbers of cells measured for each cell line to the methods and legends.

-Fig. 5: the “E” label overlaps with the axis label

We have realigned the label.

-Fig. 6C-D: some of the darker grey dots are difficult to visualise, a different colour scheme would be helpful.

The colour scheme has been tweaked to improve visibility.

-The supplementary figures need to be reorganised as in the text, they are not mentioned in order (they are mentioned as follows: S1 / S5 / S3 / S4 and there is no mention of Fig. S2).

We’ve reordered the supplemental figures and ensured that they are mentioned clearly in the correct order.

Reviewer 2 Advance Summary and Potential Significance to Field:
This MS is a submission from a group of ex-Gull lab PIs and others, and builds upon the proteome-wide localization studies. I was genuinely looking forward to reading this work as insights into the biology of trypanosomes arising from the tagging project would be of considerable interest, as would some advances in understanding organelle biogenesis. Sadly what we have here, as far as I can understand, is a poorly presented set of experiments that in many places are not well thought out, badly written and lack of obvious argumentation. The overall contribution here is limited, and I recommend reject. There seems here to be a failure to engage, with the literatire, the biology or attempts to work with the reader for clear exposition.

We are disappointed to have received a negative response, although we note that your specific comments below are often readily addressed. We have endeavoured to address your concerns; however, we found some unclear (e.g. lacking specific details or examples from the literature).

© 2022. Published by The Company of Biologists under the terms of the Creative Commons Attribution License (https://creativecommons.org/licenses/by/4.0/).

Awkward 1st line and sense of final line in abstract is incomprehensible.

We have rephrased the first and last lines of the abstract.

Page numbers on an MS are essential.

We have added line and page numbers.

Nucleolus size? Well - by what criteria - assume volume as ER is probably targets thing in terms of area, although that is membrane bound I suppose. So what anyway? Size matters…

Is this referring to the first line of the introduction? Yes, the nucleolus is the largest non-membrane bound compartment within the nucleus, and also within the cell. The ER has a membrane, so fundamentally different potential targeting mechanisms (i.e. membrane transporters, membrane anchors).

noitpecxe on si iecurb amosonapyrT etisarap ralullecinu ehT

What does this sentence mean exactly? Apologies, but cut and paste rendered this a mirror image…

By stating that *T. brucei* is no exception this emphasises that *T. brucei* is included in the preceding statement that most of eukaryotic life has a nucleus.

I am not really convinced that there is evidence as cited that the nucleolus is distinct between life stages. This seems a bit weak to me as the procyclin and ESBs are both sensu stricto extra nucleolar. I don't think this is needed as this is likely the most divergent from canonical organisms that such an analysis has been performed.

This may be true, but the evidence for the nature of a 'procyclin body' is weak. However whether or not a 'procyclin body' exists and is extra-nucleolar is not the primary issue here, the more important point is that Pol I targeting in *T. brucei* cannot be simply to the nucleolus
– It must be targeted to be to the nucleolus *and* the ESB in bloodstream forms. In procyclic forms it is more unclear, maybe the nucleolus and a procyclin body or perhaps just the nucleolus, either way different to the bloodstream.

We were trying to state this complex piece of biology in a way that is accessible to non-trypanosome biologists while being accurate, evidently failing on the latter. We have rephrased the text.

There are many NLSs, of distinct structures and forms, and a better ref would be DOI: 10.1007/s00894-017-3420-y

Which reference you think should be replaced? In any case, it is unclear why this would be a better reference for diversity of NLS. The suggested reference describes an optimised method for identifying canonical KRXR NLSs through predicted binding to α importin (the only mechanism for canonical import), not the diversity of non-canonical NLSs.

Not ALL eukaryotes, but would support MANY or WIDESPREAD

We have clarified statement to make it clear that we have provided the first strong experimental evidence from a divergent eukaryote, thus allowing us to make this statement for *diverse* eukaryotes and therefore indicating it is likely to be a feature of the last common ancestor.

F1 Resolution in B and C is poor. Also - color the different cohorts in the scatterplots?

Apologies that the resolution was poor in the version you received, the original version is high resolution, and we will check during proofing that it is readable.

© 2022. Published by The Company of Biologists under the terms of the Creative Commons Attribution License (https://creativecommons.org/licenses/by/4.0/).

Which cohorts do you mean? This data is not split into cohorts at this stage, this is simply all cell lines in which the tagged protein localised to the nucleolus, nucleoplasm and or/cytoplasm. The data points could be coloured according to their localisation category but the aim of this figure was to define a cutoff for nucleus/nucleolus enrichment relatively independent of the TrypTag annotation and not check the quality of our TrypTag annotation and have therefore left the plots without colour.

F2 Panel B the scale here needs to be linear. I cannot work out where the 30kDa would sit. Dashed lines - in some cases we are referred to one line or even none, but in all of these scattergrams there are two! It may help if one or two known nucleolar/nucleolar proteans were annotated in these plots. Panel E - what is the X-axis? Assume residue number but from what?

The scale in Figure 1B cannot practically be linear. Protein molecular weight is roughly log normally distributed, so on a non-log scale will be crushed to the left. As indicated in the main text and legend, the vertical dashed line represents cutoff of 30 kDa, but we have clarified this.

For A-C there are indeed two dashed lines on each plot. All are defined in the legend except for the vertical dashed lines in A – those represent a global intensity threshold which remained unused and should not have been visible. From their definitions, we hoped the line identify would have been self-evident from the corresponding axis names. Evidently the description was unclear, so we have rephrased the legend, and have removed the erroneous vertical lines from A.

Figure 1 has several known proteins annotated on these plots, although they were selected as nice examples of the localisation rather than their (accurate) confirmation of previous data. This includes nucleolar NOP53 (Tb927.9.13340) and nucleoplasmic DRBD4 (Tb927.11.14100). We have added gene names of these named proteins (and others) to the figure.

The X axis in Figure 2E is residue number in the motif. We have clarified this in the legend.

F3 This is just a mess - data are likely pretty good but arrangement, annotation etcetera leaves a lot to be desired and also makes the thing hard to understand. There are no axes on panel E and F or indicators of what the whiskers, boxes and dots indicate. This then becomes difficult to evaluate as I am left to assume what these data mean rather than being confident. A reviewer should not have to work herself so hard.

We are sorry that you had difficulty understanding the figure, but without clear specific comments we are not able to improve it. Eg. We are very unclear what the issue with axes is – there are clear axis titles and scales in Figure 3D, E and F. We have added additional description of the figure layout to the legend hoping that this will help.

The box plots are quartile range with the point representing the mean, as recommended by JCS guidelines. The whiskers are the 5th and 95th percentiles. We recognise that whiskers often have different definitions and these were not previously explicitly defined in the text. We apologise for omission of a clear definition and have added this to all of the relevant legends.

F4 - Does there not need to be a total cell category here? Also, I think work using LopIT and similar has identified nucleolar cohorts in several additional species - Arabidopsis certainly comes to mind and as the current taxon sampling is Opistokhonta versus a kinetoplastid, not great. This latter could presumably be improved by chosing one or two additional Kinetoplastids and analysing the relevant paralogs?

A total cell category is not particularly informative. Nuclear transport occurs from the cytoplasm, making cytoplasmic proteins the set that could be subject to nuclear import and thus the relevant comparison set. This can be alternatively stated as excluding proteins from the analysis which are inaccessible for nuclear import, for example by

© 2022. Published by The Company of Biologists under the terms of the Creative Commons Attribution License (https://creativecommons.org/licenses/by/4.0/).

being in a membrane- bound compartment (ER, glycosome, etc.), having a transmembrane domain, proteins sequestered by binding to the cytoskeleton, etc.

LOPIT/HyperLOPIT datasets have some similarities to genome-wide subcellular protein localisation data and do identify nuclear/nucleolar cohorts, however the two are not by any means equivalent. We have previously evaluated HyperLOPIT data from *Toxoplasma gondii* for this purpose (not shown in the manuscript). This dataset does not cluster mitoribosome proteins with nucleolar proteins nor chromatin-associated nuclear proteins with non- chromatin nuclear proteins. As a result, it is unclear how an equivalent analysis could be undertaken and unclear whether you'd *expect* similar results from HyperLOPIT data vs. localisation data.

Finally, a supporting analysis of paralogs seems like a circular argument – assuming I understand correctly, this proposes detecting paralogs through sequence similarity then asking whether the paralog's sequence has similar amino acids in it. The paralog will, due to having sequence similarity. In any case, if this analysis showed that, say, *Leishmania mexicana* paralogs of *T. brucei* nucleolar proteins tend to be less basic, then a confounding interpretation is that those paralogs do not localise to the nucleolus in *L. mexicana*.

I'm not very impressed with the KKKKK etcetera and RRRR etcetera strategy. As the authors state, a lot of these proteins remain in the cytosol, and a huge issue here is if these are folded, generically sticky etcetera. I'm also unclear as to if westerns have been performed here to ensure the constructs are intact and not partially degraded. It is also possible that these basic proteins are simply binding to a carrier that recruits to the nucleus for example. This is a very crude approach.

We tested an alternative strategy, dispersed positively charged residues rather than poly- K/R/KR acid runs, in Figure S5. We also tested an inverse strategy, removing negatively charged residues from nucleolar proteins, in Figure S4. In our opinion the evidence from Figure 5 is stronger than S4 and S5, hence its selection as the main figure.

We agree that a substantial proportion of the poly-K/R/KR protein remained in the cytoplasm; however, this is not unexpected as this is a candidate *nucleolar* not *nuclear* targeting mechanism. Hence, the test with the additional NLS in Figure 5C, and we have clarified the text to make this clearer.

These data certainly reflect a folded protein: We used a fluorescent protein as this self-reports its non-degraded and folded state through being fluorescent, and all cell lines clearly have strong green fluorescence. We have added a note, at the first use of these small leader sequences on mNG, to emphasise why we designed these experiments this way and have added plots of total signal intensity to show lack of degradation.

The sequences in Figure 5B may look artificial, presumably raising your 'generically sticky' concern, but there is good biological precedent. E.g. the nucleolar DEAD helicase Tb927.5.4270 has a 10 K run interrupted by a single I, NOP53 (involved in ribosome biogenesis) has a 10 K run interrupted by 1 R and 1 D. Similar runs of D, E and R also occur. Therefore, if these homo-amino acid runs are 'generically sticky' then they are likely so in a biologically relevant way. We have added these examples to the text.

We agree that basic proteins may be binding a carrier that contributes to nucleolar recruitment, this is analogous to importin α binding to the NLS for nuclear import.

The comment that the NLS improves nucleolar targeting of these probes is really not very helpful - if you provide an additional mechanism to recruit to the organelle, then perhaps this is obvious.

We did not test whether an NLS improves nucleolar targeting of these candidate nucleolar targeting sequences, that implies a comparison between e.g. mNG::KKKKKKKKKK and mNG::KKKKKKRSRE. Our title for Figure 5 was poorly phrased, which may be the source of this misunderstanding.

© 2022. Published by The Company of Biologists under the terms of the Creative Commons Attribution License (https://creativecommons.org/licenses/by/4.0/).

Instead, we tested whether a poly-K/R/KR sequence increases partition of a nuclear protein (i.e. mNG::KRSRE) to the nucleolus, which it does. We had lost the specific discussion of this result through efforts to shorten the manuscript, and have now clarified this in the results and in the Figure 5 title.

F6 - This is also well known, and simply showing this demonstrates a lack of understanding the literature.

Please state which aspect of Figure 6 or the conclusions drawn is ‘well known’. To me, one aspect of this figure, that proteins in ribonuclear machinery tend to be basic or have basic tracts, certainly is well known – however these data provide evidence for several conclusions beyond that, specifically that the mitoribosome has a different charge profile.

If there is specific literature that you think we have overlooked then please provide some relevant references, we have not found any which consider amino acid composition in this way.

Interesting that there is no citation of recent work demonstrating the divergence of the kinetoplastids mitoribosome, a spectacular story that deserves recognition here.

That work is certainly spectacular and we noted that despite an unusually adaptation as a protein-rich mitoribosome the amino acid composition of *T. brucei* is conserved in that it has the low proportion of basic amino acid similar to humans/yeast mitoribosome proteins (Figure 6A). We did not cite this originally as it is not directly relevant; however, we appreciate you drawing our attention to this as it strengthens our conclusions and we have added discussion to this effect.

Overall the work fails to meet the first criterion for acceptable work for JCS.

On reviewing the above comments, we do not think that the reviewer has given any evidence that this work is either not novel (excepting the studies in other systems we already cite and discuss) or uninteresting to a cell biology audience.

Through addressing the comments, we have provided an additional control experiments to give confidence about cell line generation, addressed concerns about data presentation where possible (although some of the concerns are unclear), and note but disagree with the dislike for some of our experimental design. None of these we believe constitute major issues.

Reviewer 2 Comments for the Author:

None

Reviewer 3 Advance Summary and Potential Significance to Field:
This manuscript presents a research work that analyzes nucleolar protein properties from the parasitic protozoa Trypanosoma brucei. The interest of this investigation is to provide additional insight to protein separation into the nucleolus. From an evolutionary point of view, to address the search of nucleolar partition determinants in an early branching organism such as T. brucei is a relevant question to pursue. The selection of proteins for this study was based on the Tryp Tag data base resource that provides cellular location of proteins encoded in the T. brucei genome, tagged and analyzed by microscopy. With this approach, the work presented provides clear evidence to support their conclusion that targeting to the nucleolus is mediated by positive charges in the nucleolar proteins, and that these charged residues aid towards a differential partition of mitochondrial and cytoplasmic ribosomal proteins. The manuscript is suitable for its publication, minor issues are mentioned below.

Thank you for your positive comments about the value of this study in providing a broader evolutionary perspective on nucleolar targeting.

© 2022. Published by The Company of Biologists under the terms of the Creative Commons Attribution License (https://creativecommons.org/licenses/by/4.0/).

Reviewer 3 Comments for the Author:

Minor issues to be addressed:

1. In legend to Figure 1 the indicator B is repeated. The second B should be changed to C.

Thank you – we have corrected this.

2. Legend to Figure S2: A-C and A. B. C. Legend states that the mis-localized fusion proteins are the ones tagged on the N-terminus. Nevertheless, as shown in the corresponding images, the mis-localized proteins at those tagged on the C-terminus. The legend should be corrected in all 4 cases.

You are absolutely correct, we have corrected this too.

3. A statement regarding the multifunctionality of the nucleolus may be included to improve the manuscript.

We have added a short note emphasising that ribosome production is not the only function of the nucleolus.

Second decision letter

MS ID#: JOCES/2021/259701

MS TITLE: Nucleolar targeting in an early-branching eukaryote suggests a general mechanism for ribosome protein sorting

AUTHORS: Milad Jeilani, Karen Billington, Jack Daniel Sunter, Samuel Dean, and Richard John Wheeler
ARTICLE TYPE: Research Article

I am happy to tell you that your manuscript has been accepted for publication in Journal of Cell Science, pending standard ethics checks.

© 2022. Published by The Company of Biologists under the terms of the Creative Commons Attribution License (https://creativecommons.org/licenses/by/4.0/).
